# Diagnostic and prognostic significance of transient ischemic dilation (TID) in myocardial perfusion imaging: A systematic review and meta-analysis

**DOI:** 10.1007/s12350-017-1040-7

**Published:** 2017-09-25

**Authors:** Mohamed Alama, Christopher Labos, Handel Emery, Robert M. Iwanochko, Michael Freeman, Mansoor Husain, Douglas S. Lee

**Affiliations:** 1 Division of Cardiology, Peter Munk Cardiac Center and the Joint Department of Medical Imaging, Toronto, Canada; 2 Institute for Clinical Evaluative Sciences, Toronto, Canada; 3 University of the West Indies, Kingston, Jamaica; 4 St. Michael’s Hospital, Toronto, Canada; 5 Department of Medicine, University of Toronto, Toronto, Canada; 6 Ted Rogers Centre for Heart Research, Toronto, Canada

**Keywords:** SPECT, outcomes research, diagnostic and prognostic application

## Abstract

**Background:**

Transient ischemic dilatation (TID) of the left ventricle is a potential marker of high risk obstructive coronary artery disease on stress myocardial perfusion imaging (MPI). There is, however, interstudy variation in the diagnostic performance of TID for identification of severe and extensive coronary disease anatomy, and varied prognostic implications in the published literature.

**Methods:**

We searched MEDLINE, EMBASE, and COCHRANE databases for studies where TID was compared with invasive or CT coronary angiography for evaluation of coronary artery stenosis. Two reviewers independently evaluated and abstracted data from each study. A bivariate random effects model was used to derive pooled sensitivities and specificities, in order to account for correlation between TID in MPI and anatomic disease severity.

**Results:**

A total of 525 articles were reviewed, of which 51 met inclusion criteria. Thirty-one studies contributed to the analysis, representing a total of 2037 patients in the diagnostic meta-analysis and 9003 patients in the review of prognosis. The ratio above which TID was deemed present ranged from 1.13 to 1.38. Pooled sensitivity was 44% (95% CI 30%-60%) and specificity was 88% (95% CI 83%-92%) for the detection of extensive or severe anatomic coronary artery disease. Analysis of outcome data demonstrated increased cardiac event rates in patients with TID and an abnormal MPI. In otherwise normal perfusion, TID is an indicator of poor prognosis in patients with diabetes and/or a history of coronary disease.

**Conclusions:**

Among patients undergoing MPI, the presence of TID is specific for the detection of extensive or severe coronary artery disease.

**Electronic supplementary material:**

The online version of this article (doi:10.1007/s12350-017-1040-7) contains supplementary material, which is available to authorized users.

## Spanish Abstract

**Antecedentes:**

La Dilatación Isquémica Transitoria (TID) del ventrículo izquierdo es un marcador potencial de enfermedad arterial coronaria obstructiva de alto riesgo en la fase de estrés del estudio de perfusión miocárdica (EPM). Existe, sin embargo, una variación entre los estudios en el rendimiento diagnóstico de la TID para identificar la severidad y extensión de la enfermedad coronaria anatómica, así como diversas implicaciones pronosticas en la literatura publicadas.

**Métodos:**

Buscamos en las bases de datos de MEDLINE, EMBASE y COCHRANE estudios donde el TID fue comparado con la angiografía coronaria invasiva o por TC (Tomografía Computada) para la evaluación de la estenosis arterial coronaria. Dos revisores evaluaron de forma independiente resumieron y evaluaron los datos de cada estudio. Se utilizo un modelo de efectos aleatorios bivariante para derivar las sensibilidades y especificidades combinadas, a fin de obtener la correlación entre la TID en el EPM y la severidad de la enfermedad anatómica.

**Resultados:**

Se revisaron un total de 525 artículos, de los cuales 51 cumplieron el criterio de inclusión. Treinta y un estudios contribuyeron al análisis, representando un total de 2037 pacientes para el meta-análisis diagnóstico y 9003 pacientes para la revisión del pronóstico. La proporción por encima de la cual la TID se consideró presente osciló entre 1.13 y 1.38. La sensibilidad combinada fue del 44% (IC del 95%: 30-60%) y la especificidad fue del 88% (IC del 95%: 83-92%) para la detección de enfermedad coronaria anatómica extensa o grave. El análisis de los datos de resultado demostró un aumento de las tasas de eventos cardíacos en pacientes con TID y un EPM anormal. En la perfusión normal, la TID es un indicador de mal pronóstico en pacientes con diabetes y/o antecedentes de enfermedad coronaria.

**Conclusiones:**

En pacientes que son llevados a un EPM, la presencia de TID es especifico para la detección de enfermedad arterial coronaria extensa o severa.

## Chinese Abstract

**背景:**

负荷心肌灌注显像 (MPI) 下, 左心室短暂缺血性扩张 (TID) 是高风险冠心病的一个潜在指标。然而, 现有的文献对于 TID 诊断严重冠心病以及评价预后的报道不尽相同。

**方法:**

我们利用 MEDLINE, EMBASE 和 COCHRANE 数据库, 检索了 TID 与冠脉造影或 CT 对比来评估冠状动脉狭窄的文献。两个评审者独立的从每个研究中评估和提取数据。为了了解 TID 和冠状动脉狭窄之间的关联性, 用一个二元随机效应模型来获得合并的敏感性和特异性。

**结果:**

总归回顾了 525 篇文献, 其中 51 篇符合入选标准。 31 个研究有助于本文的分析, 在诊断性分析中共 2037 个病人, 预后分析中共 9003 病人。TID 定义为比值超过 1.13~1.38。此时, 诊断严重冠心病的合并敏感性是 44% (95%CI; 30-60%), 特异性是 88% (95%CI; 83-92%)。在 MPI 异常的患者中, TID 能够增加心血管事件的发生率; 在 MPI 正常, 有糖尿病和/或冠心病的患者中, TID 也是一个独立的预后指标。

**结论:**

在 MPI 患者中, TID 是一个诊断严重冠心病以及评价预后的特异性指标。

## French Abstract

**Contexte:**

La dilatation ischémique transitoire (DIT) du ventricule gauche mesurée par scintigraphie de perfusion myocardique est un factor pronostique relativement important dans l’évaluation des risques liés à la maladie coronarienne obstructive. Néanmoins les protocoles d’évaluation de la DIT et la signification des résultats obtenus dans la maladie coronarienne sévère sont assez variables dans les études publiées dans la littérature.

**Méthodes:**

Dans cet article nous avons recherché dans les bases de données MEDLINE, EMBASE et COCHRANE les études où les résultats de la DIT ont été comparés à une angiographie coronaire obtenue de manière invasive ou par tomodensitométrie. Deux experts ont extrait et évalué indépendamment les données de chaque étude. De manière à tenir compte de la corrélation entre la DIT et la sévérité de la maladie coronaire une analyse bi-variée des effets aléatoires a été utilisée pour déterminer la sensibilité et la spécificité globales.

**Résultats:**

Au total, 525 articles ont été examinés, dont 51 répondant aux critères d’inclusion. Trente et une études ont contribué à l’analyse, incluant un total de 2037 patients dans la méta-analyse diagnostique et 9003 patients dans l’examen du pronostic. Le rapport au-dessus duquel la DIT a été jugé anormal varie de 1,13 à 1,38. La sensibilité globale est de 44% (IC à 95% ; 30 à 60%) et la spécificité globale est de 88% (IC 95%; 83-92%) pour la détection d’une maladie coronaire étendue ou sévère. Une augmentation du nombre d’événements cardiaques est présente chez les patients démontrant une DIT avec étude anormale de perfusion myocardique. Lorsque la perfusion myocardique est normale, une DIT est également un facteur de mauvais pronostic chez les patients atteints de diabète et/ou d’un antécédent de maladie coronarienne.

**Conclusions:**

La présence d’une DIT obtenue par scintigraphie myocardique est spécifique pour la détection de la maladie coronaire étendue ou sévère.

## Introduction

Myocardial perfusion Imaging (MPI) is an established tool for the diagnosis and risk stratification of patients with coronary artery disease (CAD) for over three decades.^1^ MPI has excellent diagnostic and prognostic accuracy and also provides good insight into cardiac function through the interpretation of a variety of perfusion and functional parameters.^2^,^3^ One of these functional parameters is transient ischemic dilation (TID), which has been validated both as a marker of severe and extensive coronary artery disease and as a predictor of cardiac outcomes in independent studies.^1^-^3^

To date, the pathophysiology of ischemic LV dilatation remains unclear with the theory of subendocardial ischemia gaining the widest acceptance.^4^,^5^ Others cite data supporting ischemia induced physical LV dilation post stress.^6^,^7^ However, several studies have demonstrated that ischemic LV dilatation may be present in patients with normal perfusion and no significant epicardial coronary disease; for example in patients with hypertrophic cardiomyopathy,^8^ or in patients with hypertensive heart disease and left ventricular hypertrophy.^9^ Therefore the true diagnostic accuracy of TID on MPI is debated and the optimal threshold for its definition remains undefined.

In this study, we conducted a meta-analysis of the diagnostic performance of the presence of TID, compared to anatomical coronary artery assessment. We also conducted a systematic review of the prognostic significance of TID. Both components of our study included patients who underwent either exercise or pharmacologic stress MPI.

## Methods

We employed a systematic search of the MEDLINE, EMBASE, and COCHRANE databases. We searched for English language studies, which examined the diagnostic and/or prognostic accuracy of TID in myocardial perfusion imaging. The search words used were (transient ischemic dilation, transient ischaemic dilation, left ventricular dilation, transient dilation, SPECT, single photon tomography, CT single photon, myocardial perfusion imaging, and myocardial scintigraphy).

Two investigators (MA, HE) independently reviewed the studies and extracted the relevant data including patient demographics, the radiotracer used, the stress modality, and findings on coronary angiography. Where additional data were required to complete the meta-analysis or discrepancies existed, attempts were made to contact the original authors to obtain such information. We excluded studies where: a) there was no coronary arterial anatomic assessment (either invasive coronary angiogram or coronary CT angiography) for comparison, or b) there was no clear documentation of the method used to calculate the ratio above which TID was diagnosed.

The studies identified for inclusion by the two investigators, and data extracted, were reviewed for eligibility and accuracy by a third investigator (DSL). Methodological quality regarding the risk of bias and concerns of applicability was assessed using the Quality Assessment of Diagnostic Accuracy Studies 2 (QUADAS 2) tool.^10^ QUADAS 2 is a tool used for quality assessment of diagnostic accuracy studies included in systematic reviews and meta-analyses, to assess the risk of bias and applicability for use in systematic reviews. This tool contains 4 domains: patient selection, index test, reference standard, and flow and timing.

### Statistical Analysis

Data were extracted to construct 2 × 2 tables, from which the sensitivity and specificity of each study was calculated. The sensitivity and specificity estimates were pooled using a bivariate random effects model, as recommended by the Cochrane Diagnostic Test Accuracy Working Group.^11^

The bivariate model was then used to construct a hierarchical summary receiver operator curve (ROC). A *P* value <.05 was considered statistically significant. We did not calculate an I^2^ statistic given that it is not an accepted method of measuring heterogeneity between diagnostic studies.^11^ Univariate meta-regression was used to assess the significance of key covariates that were likely to affect the diagnostic accuracy of the test. Due to the small number of studies, multivariate meta-regression was not performed as it was likely to be underpowered to detect any differences.

All statistical analyses were conducted using STATA/SE, version 12.0 (Stata Corp LP, College Station, Texas, USA).

## Results

### Summary of Studies Examining TID

From the initial database search, we identified 525 citations of which 368 articles remained after removing duplicates. After reviewing the titles and abstracts of these records, 317 articles were excluded because they were not relevant to the purpose of the study.

**Figure 1:**
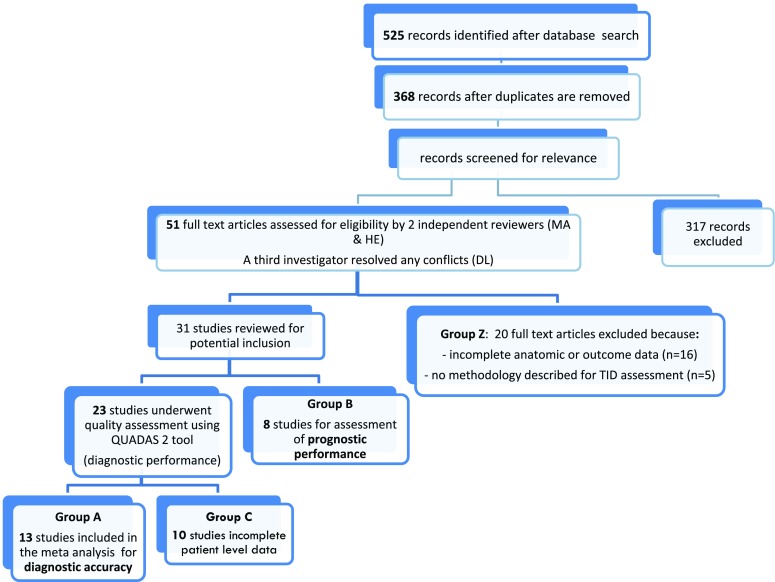
Search strategy and study selection

Of the remaining 51 articles, 20 studies were excluded because: (a) there was no evaluation of coronary anatomy (invasive coronary angiogram or CT angiography) for diagnostic studies [n = 11], (b) they included only patients with left ventricular dysfunction and fixed perfusion defects for prognostic studies [n = 1], (c) there was no clear documentation of the method used to calculate the ratio above which TID was diagnosed or only visual assessment of TID was employed [n = 5], (d) incomplete data [n = 2], and (e) duplicate data [n = 1] as shown in Figure 1 (Group Z). These excluded studies are shown in Online Table A. We included 31 studies, of which 23 evaluated TID from a diagnostic perspective. Of these, 13 studies were included in the quantitative meta-analysis (Figure 1, Group A), and 10 studies were not quantitatively synthesized because the patient-level data were only reported in aggregate, and patients could not be separated into TID positive or negative, or severe or non-severe CAD categories (Group C). The quantitative meta-analysis encompassed a total of 2037 patients in the diagnostic evaluation and 9003 patients in the prognostic evaluation (Figure 1). There were 8 studies examining the prognostic significance of TID, which did not report patient-level data and therefore were incorporated in a narrative synthesis (Group B).

### Studies of Diagnostic Performance

**Figure 2:**
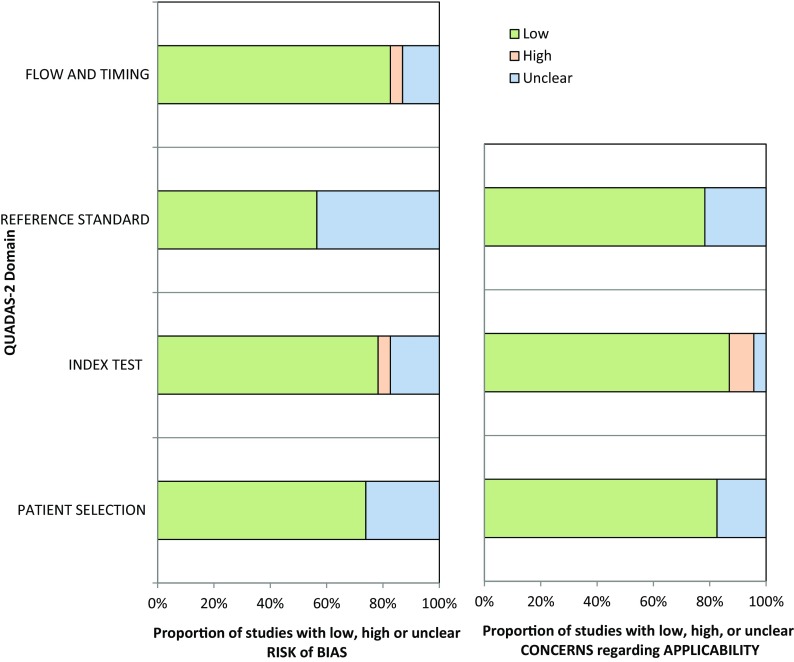
Quality assessment using QUADAS-2

Characteristics of the 13 studies included in the quantitative meta-analysis are shown in Table 1, and the 10 non-quantitatively analyzed studies are shown in Online Table B. The definition of severe and extensive CAD and software used for TID evaluation are shown in Table 2. Technetium-99 was the most commonly utilized radiotracer (8 studies), followed by Tl-201 (2 studies), dual isotope scanning (2 studies), and Rb-82 (1 study). Coronary angiography was performed in 73% of patients. The modality of stress included exercise (4 studies), pharmacologic stress (6 studies), and both exercise and pharmacologic stress (3 studies). Methodological Quality Assessment using the QUADAS 2 tool revealed that most of the studies included in the meta-analysis demonstrated low risk for bias or concern regarding applicability (Figure 2).

**Table 1:** Studies included in the meta-analysis of diagnostic performance of TID

Study	Stress/tracer	TID ratio (quant or qual)	# SPECT vs coronary angiography or CTA	Cardiac risk factors	Cardiac characteristics
Xu et al^28^ 62.4 ± 10.0 years 62% men	Exercise Tc 99m	1.19 (quant)	SPECT: 547 pts, angiography or CTA: 215 pts	Smoking 15%, HTN 59%, DM 18%, Dyslipidemia 50%	Angina 17%
Kinoshita et al^29^ 63.1 ± 7.8 years 87% men	Exercise Tc 99m	1.012 (quant)	SPECT: 75 pts, angiography or CTA: 55 pts	N/A	N/A
Petretta et al^30^ 63 ± 9 years 75% men	Ex and Pharm Tc 99m	1.19 (quant)	SPECT: 692 pts, angiography or CTA: 242 pts	Smoking 47%, HTN 88%, DM 100%, Dyslipidemia 75%	Angina 31%, MI 16%
Weis et al^1^ 58 years 78% men	Exercise Tl 201	1.12 (quant and qual)	SPECT: 89 pts, angiography or CTA: 89 pts	N/A	N/A
Chouraqui et al^6^ 67 ± 3 years 47% men	Pharm Tl 201	1.12 (quant)	SPECT: 73 pts, angiography or CTA: 73 pts	N/A	MI 33%
Emmett et al^31^ 68 ± 10 years 64% men	Adenosine Tc 99m	1.19 (quant)	SPECT: 175 pts, angiography or CTA: 55 pts	HTN 82%, DM 81%	N/A
Fallahi et al^18^ 56 ± 11 years 23% men	Pharm Tc 99m	1.17 (quant)	SPECT: 86 pts, angiography or CTA: 38 pts	Smoking 17%, HTN 55%, Dyslipidemia 36%	N/A
Emmett et al^32^ 72 ± 8 years, 47% men	Ex and Pharm Tc 99m	1.22 (quant and qual)	SPECT: 103 pts, angiography or CTA: 103 pts	DM 58%	N/A
Marcassa et al^5^ 61 ± 8 years % men N/A	Ex and Pharm Tc 99m	1.24 (quant)	SPECT: 234 pts, angiography or CTA: 186 pts	HTN 33%, DM 5%, Dyslipidemia 42%	Angina 47%, MI 63%
Rischpler et al^27^ 64 ± 11 years 58% men	Pharm Rb 82	1.13 (quant)	SPECT: 265 pts, angiography or CTA: 81 pts	Smoking 26%, HTN 84%, DM 37%, Dyslipidemia 58%	MI 26%, PCI 42%
Mazzanti et al^33^ 66 ± 11 years % men N/A	Exercise Dual	1.22 (quant)	SPECT: 228 pts, angiography or CTA: 174 pts	N/A	MI 35%, CABG 33%
Abidov et al^34^ 69 ± 11.4 years 54% men	Pharm Dual	1.36 (quant)	SPECT: 356 pts, angiography or CTA: 179 pts	HTN 70%, DM 31%	Angina 48%
Golzar et al^12^ 62 ± 13 50% men (validation group)	Pharm Tc 99m	1.31 (quant)	SPECT: 647 pts, angiography or CTA: 547 pts	HTN 86%, Dyslipidemia 63%	Chest pain 54%

*TID*, transient ischemic dilation; *CTA*, CT angiography; *Pts*, patients; *HTN*, hypertension; *DM*, diabetes; *N/A*, not applicable; *MI*, myocardial infarction; *PCI*, percutaneous coronary intervention; *CABG*, coronary artery bypass graft

**Table 2:** Definition of CAD severity and software used for TID calculation

Study	Definition of severe and extensive CAD	Software used for TID calculation
Xu et al^28^	≥70% LM or proximal LAD or ≥90% of ≥2 coronary vessels	Cedars Sinai QPS and QGS software
Kinoshita et al^29^	≥75% reduction of luminal diameter of ≥2 coronary vessels	GMS-550 A Toshiba workstation Formula used for calculation (Mean A EX/Mean A rest)
Petretta et al^30^	≥70% proximal LAD or ≥90% of ≥2 coronary vessels	Cedars Sinai software
Weis et al^1^	≥90% of ≥2 coronary vessels	Manual calculation form the anterior view planar images
Chouraqui et al^6^	≥90% of 3 coronary vessels	Manual calculation form the anterior view planar images
Emmett et al^31^	≥90% of LAD or ≥90% of ≥2 coronary vessels	Emory Cardiac toolbox
Fallahi et al^18^	Coronary artery index Gensini score used	Cedars Sinai Auto Quant software package
Emmett et al^32^	≥90% of LAD or ≥90% of ≥2 coronary vessels	Emory Cardiac toolbox
Marcassa et al^5^	>50% diameter stenosis in major pericardial vessel modified Gensini score	Not specified
Rischpler et al^27^	Obstructive CAD (Cath/CTA) severity not specified	Card IQ Physio (GE healthcare)
Mazzanti et al^33^	≥90% of proximal LAD or ≥90% of ≥2 coronary vessels	Cedars Sinai QPS and QGS software
Abidov et al^34^	≥90% of proximal LAD or ≥90% ≥2 vessel disease	Cedars Sinai QPS software
Golzar et al^12^	≥70% LM or proximal LAD or ≥90% of ≥2 coronary vessels	4DM-SPECT version 5.1

*CAD*, coronary artery disease; *TID*, transient ischemic dilation; *LM*, left main; *LAD*, left anterior descending; *Cath*, cardiac catheterization; *CTA*, CT angiography

**Figure 3:**
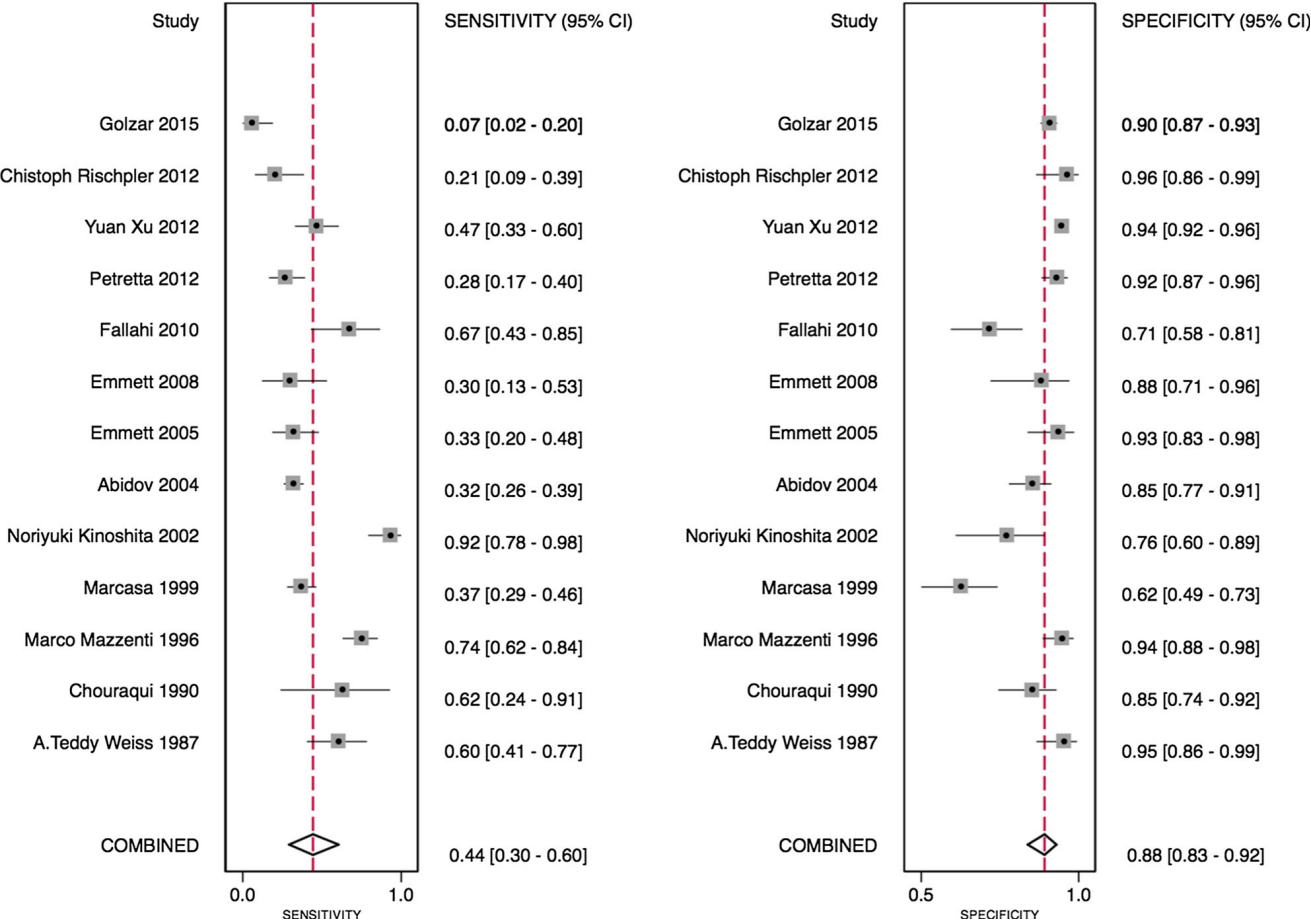
Forest plot of included studies in diagnostic meta-analysis

**Figure 4:**
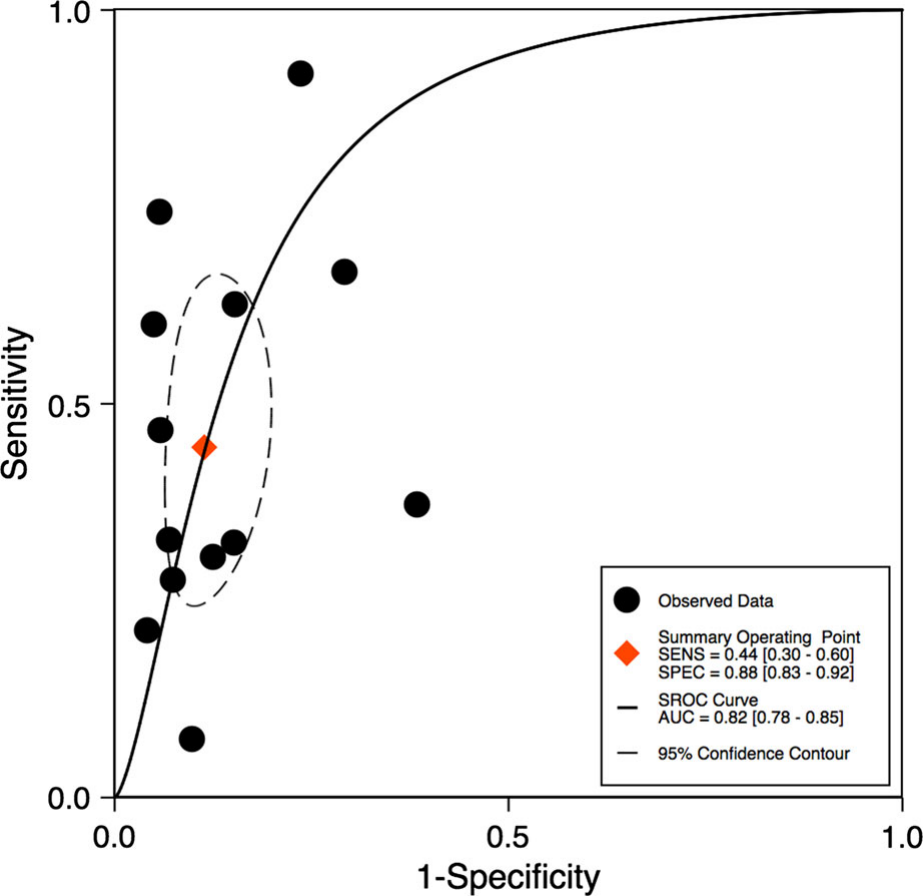
ROC curve for all studies

The ratio above which TID was diagnosed ranged from 1.13 to 1.38, with differences noted depending on the tracer used. Using the published TID ratios (as defined by the individual study authors), the sensitivity of TID for the detection of extensive or severe CAD ranged from 21% to 62.5% (see Table 3). One study only showed very low sensitivity (7%).^12^ Specificities were higher and demonstrated less variability, ranging from 77% to 98% (Table 3). Bivariate analysis of the 13 studies, which had complete statistical data, revealed a pooled sensitivity of 44% (95% confidence interval [CI] 30%-60%) and a pooled specificity of 88% (95% CI 83%-92%) as shown in Figure 3. The pooled area under the ROC curve was 0.82 (0.78-0.85) for all studies (Figure 4).

**Table 3:** Diagnostic performance of TID in meta-analyzed studies

Study	Sensitivity	Specificity	PPV	NPV
Xu et al^28^	56	90	56	90
Kinoshita et al^29^	91.4	76.9	79	90
Petretta et al^30^	27.5	92.9	59.3	76
Weis et al^1^	60	95	85	82
Chouraqui et al^6^	62.5	85	–	–
Emmett et al^31^	30.4	87.5	63.6	63.6
Fallahi et al^18^	66.7	70.8	–	–
Emmett et al^32^	30	93	79	63
Marcassa et al^5^	37	61	64	34
Rischpler et al^27^	21	95.8	77	63
Mazzanti et al^33^	77	92	–	–
Abidov et al^34^	73	88	–	–
Golzar et al^12^	7	98	–	–

### Subgroup Analyses for Diagnostic Performance of TID

**Figure 5:**
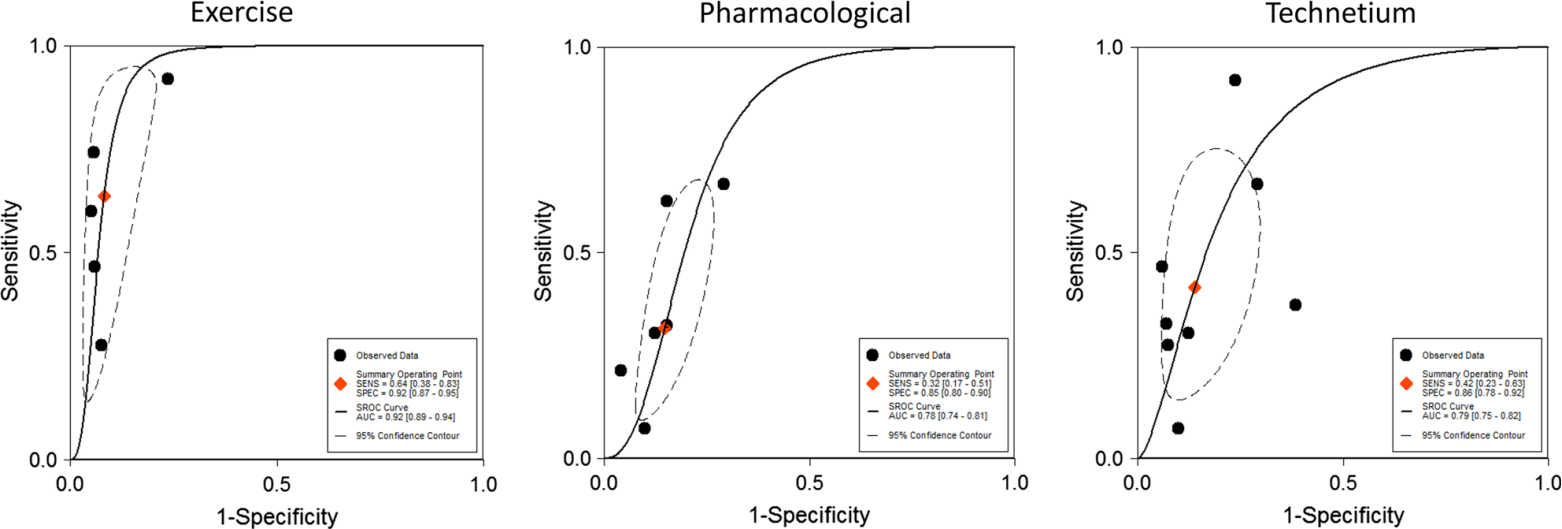
ROC curves in different subgroups

Subgroup analysis of the technetium studies revealed a pooled sensitivity of 42% (95% CI 23%-63%) and a pooled specificity of 86% (95% CI 78%-92%), which was similar to the overall results above (see Figure 5). Subgroup analyses also demonstrated that studies using exercise as a stressor demonstrated a significantly higher pooled area under the receiver operating characteristic curve (AUC 0.92 vs 0.78, *P* < .001) for detection of severe CAD compared to studies using pharmacological stressors (Figure 5). In studies that determined the presence of TID qualitatively (instead of quantitatively) the pooled sensitivity was 46% (95% CI 38%-54%) and pooled specificity was 88% (95% CI 79%-93%).

For studies using technetium, the TID range was 1.13-1.31. In these studies, the sensitivity ranged from 34% to 56%, while the specificity ranged from 77% to 98% for detection of severe CAD. Overall sensitivity and specificity were similar for both exercise and pharmacologic technetium studies. Based on univariate meta-regression, the TID ratio cutoff used in various studies had little effect on specificity (*P* = .99). However, higher TID ratios resulted in decreased sensitivity (*P* < .01).

### Studies of Prognostic Performance

Studies evaluating TID as a marker of increased cardiac events are summarized in Table 4. Across studies, the annualized rates of cardiac death or MI ranged from approximately 0.2% to 1% in those with no TID, 2% to 5% in those with TID and normal perfusion, and 5% to 6% among those with TID and ischemia, CAD, or diabetes. De Winter reported that TID was a significant predictor of all-cause mortality even after multivariable adjustment for resting heart rate, beta-adrenoreceptor antagonism, summed rest score, and resting LV ejection fraction.^13^

**Table 4:** Studies included in the systematic review of prognosis

Study	Demographics and follow-up	TID ratio (quant or qual), n(%) of pts with TID	Annualized cardiac outcomes
Abidov et al^17^ 66.1 ± 12.8 years 50% men Ex and Pharm Dual	Primary study cohort: 1560 pts with normal perfusion; Secondary cohort: 2037 pts with normal or near-normal perfusion: 2.3 ± 0.7 years	1.21 (quant) n = 390 (25%)	Outcome: cardiac death, MI, or coronary revascularization Normal perfusion (SSS = 0): (a) TID present: 2.4%·year^−1^; (b) No TID (i.e., lowest TID quartile): 0.6%·year^−1^ Normal/near-normal perfusion (SSS 0-3): (a) TID present: 2.2%·year^−1^; (b) Other 3 TID quartiles: 1.0%·year^−1^
Petretta et al^14^ 64 ± 8 years 67% men Ex and Pharm Tc 99m	672 pts with DM 2+ years	1.19 (quant) n = 97 (14%)	Outcome: cardiac death or MI No ischemia + LVEF > 45%: (a) TID present: 4.9%·year^−1^ (b) No TID: 0.21%·year^−1^ Ischemia present + LVEF > 45%: (a) TID present: 5.4%·year^−1^ (b) No TID: 1.9%·year^−1^ Ischemia + LVEF ≤ 45%: (a) TID present: 7.2%·year^−1^ (b) No TID: 3.8%·year^−1^
Doukky et al^19^ 58 ± 12 years 52% men Ex and Pharm Tc 99m	1236 pts with normal perfusion 27 ± 9 (clinical events) 37 ± 8 months (mortality)	1.16 (Ex) 1.22 (Pharm) (quant) n = 76 (6%)	Outcome: cardiac death or MI Overall: (a) TID present: 2.4%·year^−1^ (b) No TID: 0.4%·year^−1^ DM or CAD: (a) TID present: 5.9%·year^−1^ (b) No TID: 0.2%·year^−1^
Uz zaman et al^15^ 56 ± 9 years 67% men Ex and Pharm Tc 99m	2689 pts in a single-center 18 ± 4 months	1.22 (quant) n = 189 (7%)	Outcome: fatal MI (a) TID + not revascularized: 3.3%·year^−1^ (b) TID + revascularized: 1.6%·year^−1^ (c) No TID: not reported Outcome: non-fatal MI (a) TID + not revascularized: 14.2%·year^−1^ (b) TID + revascularized: 3.3%·year^−1^ (c) No TID: not reported
Lette et al^16^ 61 ± 10 years 55% men Pharm Tl 201	510 total pts; Prognostic cohort: 187 undergoing non-cardiac surgery 12 months	1.15 (quant) n = 45 (9%)	Outcome: cardiac death or MI (a) TID present: 58%·year^−1^ (b) Reversible defect: 19%·year^−1^ (c) Normal: 2%·year^−1^
Lette et al^35^ 62 years 57% men Pharm Tl 201	753 pts 16 months	1.15 (quant) n = 41 (5%)	Outcome: cardiac death or MI (a) TID present: 47.4%·year^−1^ (b) Reversible defect: 15.8%·year^−1^ (c) Normal: 2.0%·year^−1^
Thomas et al^36^ 65 ± 12 years 62% men Ex and Pharm Tc 99m and Dual	1612 pts 24 ± 7 months	Qualitative n = 371 (23%)	Outcome: cardiac death or MI Technetium: (a) TID present: 6%·year^−1^ (b) No TID: 1.5%·year^−1^ Dual isotope: (a) TID present: 3.8%·year^−1^ (b) No TID: 0.5%·year^−1^
De winter et al^13^ 78 (IQR: 76-80) years 54% men Ex and Pharm Tc 99m	294 pts aged ≥ 75 years old 26 months	1.005 (quant) n = 147	Outcome: all-cause death (a) TID present: 9.7%·year^−1^ (b) No TID: 5.0%·year^−1^

*TID*, transient ischemic dilation; *Ex*, exercise stress; *Pharm*, pharmacologic stress; *Dual*, dual isotope study; *MI*, myocardial infarction; *SSS*, summed stress score; *DM*, diabetes; *LVEF*, left ventricular ejection fraction; *CAD*, coronary artery disease; *Pts*, patients; *IQR*, interquartile range

There was heterogeneity of patients in the different prognostic studies of TID. In a study of diabetic patients with TID and ischemia by Petretta et al, the annualized rate of cardiac death or non-fatal MI was 7.2% with post-stress LVEF ≤45% and 5.4% when post-stress LVEF was greater than 45%.^14^ In another study of patients with TID, rates of fatal and non-fatal MI were substantially increased in those who were not revascularized, compared to those who underwent CABG surgery or PCI.^15^ One study examined patients undergoing MPI prior to non-cardiac surgery and reported high postoperative cardiac event rates: 58% in the presence of TID, 19% with reversible perfusion defects but no TID, and 2% in patients with normal scans. These events were temporally accelerated, with the majority of cardiac events occurring within 4 months postoperatively.^16^

### Special Consideration of TID in Patients with Otherwise Normal Perfusion Scans

The study by Abidov et al demonstrated that TID is an independent prognostic marker for cardiac events in patients with either normal or near-normal MPI.^17^ Patients in the highest TID quartile (mean TID ratio of 1.35 ± 0.14) were older and diabetic. The prognostic impact of TID with normal myocardial perfusion was modified by the presence of CAD or diabetes,^18^ with an increased risk of cardiac death or MI reported in these patients.^19^ In a study of diabetes patients with normal post-stress LVEF and no ischemia, the annual event rate was 4.9% in those with TID and 0.2% in those without TID (*P* < .001).^14^

## Discussion

Our study showed that TID is a specific but not a sensitive marker for detection of severe and extensive CAD with a pooled sensitivity of 44% and pooled specificity of 88%. In the analysis of subgroups, we found that exercise stress resulted in a trend toward higher sensitivity than pharmacologic stress, but specificity was similar. The prognostic studies demonstrated consistently elevated risk when TID was present despite somewhat different populations studied.

This risk was heightened in those with TID and post-stress LVEF ≤ 45%, with rates of cardiac death or MI exceeding 7%·year^−1^. Among patients with normal perfusion scans, the presence of TID was associated with increased risk primarily when patients had a history of CAD or DM.

While there are many disparate studies examining diagnostic test performance, few have utilized meta-analytic approaches and summary receiver operating characteristic curves to evaluate the performance of a specific high risk marker such as the presence of TID. Many systematic reviews and meta-analyses were conducted to study the overall diagnostic and prognostic role of different imaging modalities like stress echocardiography,^20^ cardiac PET,^21^ and coronary CT angiography.^22^,^23^ However, meta-analyses of specific components of a diagnostic test, such as TID are less commonly encountered. Despite the importance that is imparted to the presence of TID in nuclear cardiology, to our knowledge, this is the first meta-analysis that studied its quantitative diagnostic performance for detection of severe CAD and prognostic performance for prediction of cardiac outcomes.

Mechanistic studies may explain, in part, the reason for the high specificity and low sensitivity of TID. Prior studies have demonstrated that TID results from subendocardial ischemia with apparent LV dilatation due to decrease in the radiotracer uptake in the endocardium.^4^,^5^ Others have proposed that TID is a manifestation of LV dilatation post-stress due to ventricular dysfunction.^24^ Therefore, the presence of TID usually indicates the existence of severe CAD, favoring higher specificity and lower sensitivity for less-critical or less-extensive disease. The diagnostic performance of TID could also be impacted because it may occur in those with hypertensive heart disease, hypertrophic cardiomyopathy, and in some patients undergoing 2-day protocols, with concomitantly normal epicardial coronary vessels.^8^,^9^

TID in myocardial perfusion imaging has been proposed as a diagnostic and prognostic marker for the detection of severe and extensive CAD; however, there is variability in the literature on its utility. To our knowledge, this is the first meta-analysis to: (a) examine the range of different ratios above which TID was diagnosed, (b) systematically review the pooled diagnostic performance of TID, and (c) examine the value of TID as a prognostic tool in a systematic review. Our meta-analysis confirms the usefulness of TID in myocardial perfusion imaging as a high risk marker for stress induced myocardial ischemia and its ability to predict future cardiac events. Based on our findings, we propose a modified algorithm approach^25^ in the presence of TID. Clearly, if both TID and high risk MPI are present, consideration should be given to invasive coronary angiography.^25^ However, since specificity of TID is high, if TID and non-high risk MPI (SSS < 4) are present in a patient with an intermediate clinical risk (CAD, diabetes, or chronic kidney disease), further non-invasive evaluation may be beneficial.^25^ Finally, the methodology of quantitative meta-analysis to evaluate other putative high risk diagnostic markers nested within imaging modalities,^26^ may be useful in future cardiac imaging research.

While meta-analyses are valuable tools for synthesizing the published literature, there are always limitations to such analyses. For example, the different patient populations, techniques, and diagnostic cutoffs all contribute to the clinical heterogeneity of the published literature. While higher TID ratio did affect sensitivity, meta-regression did not identify any other clinical variable that affected the effect estimates. However, due to the relatively small number of publications with interstudy differences in stress modality and tracer employed, the impacts of these factors may have been underrepresented. The majority of studies reported the definition of angiographic severity of coronary artery disease using percent stenosis, except the study by Rischpler.^27^ We included this study because there were high rates of prior cardiac history (e.g., prior MI, documented CAD, prior coronary revascularization procedures) in the majority of patients and it was the only study that utilized Rb PET.^27^ However, we did do a sensitivity analysis excluding this study and it revealed that there was no significant change in the diagnostic performance of TID after exclusion of this particular study, with a pooled sensitivity of 47% (31%-63%), specificity of 88% (82%-92%), and an AUC of 0.82 (0.78-0.85).

## New Knowledge Gained

The presence of TID has a high pooled area under the receiver operating characteristic curve for the detection of severe and extensive CAD. While sensitivity is low, specificity of TID is high for the detection of severe and extensive CAD.

The rate of cardiac death or MI is increased in those with TID and normal perfusion, primarily amongst those with DM, CAD, or ischemia. Rates of cardiac death or MI appear to be increased further in those with reduced LVEF.

## Conclusion

In conclusion, in this meta-analysis, we found that transient ischemic dilation during myocardial perfusion imaging is a specific diagnostic marker of severe and extensive coronary artery disease. Transient ischemic dilation is an indicator of poor prognosis, and risks were significantly elevated among those with evidence suggestive of coronary disease or reduced stress LV ejection fraction. The presence of TID significantly worsens prognosis even among diabetes patients with normal perfusion. Therefore, TID should be considered a high risk marker that may guide clinical management in patients with suspected or known coronary artery disease.

## Electronic supplementary material

Below is the link to the electronic supplementary material.

Supplementary material 1 (DOCX 137 kb)

Supplementary material 2 (PPTX 4413 kb)

## Abbreviations

CABG: Coronary artery bypass graft
CAD: Coronary artery disease
CI: Confidence interval
CT: Computed tomography
DM: Diabetes mellitus
LVEF: Left ventricular ejection fraction
MI: Myocardial infarction
PCI: Percutaneous coronary intervention
ROC: Receiver operating characteristic
TID: Transient ischemic dilation

## References

[1] Weiss AT, Berman DS, Lew AS, Nielsen J, Potkin B, Swan HJ (1987). Transient ischemic dilation of the left ventricle on stress thallium-201 scintigraphy: A marker of severe and extensive coronary artery disease. J Am Coll Cardiol.

[2] Iskandrian AS, Heo J, Nguyen T, Lyons E, Paugh E (1990). Left ventricular dilatation and pulmonary thallium uptake after single-photon emission computer tomography using thallium-201 during adenosine-induced coronary hyperemia. Am J Cardiol.

[3] Bestetti A, Di Leo C, Alessi A, Triulzi A, Tagliabue L, Tarolo GL (2001). Post-stress end-systolic left ventricular dilation: A marker of endocardial post-ischemic stunning. Nucl Med Commun.

[4] Takeishi Y, Tono-oka I, Ikeda K, Komatani A, Tsuiki K, Yasui S (1991). Dilatation of the left ventricular cavity on dipyridamole thallium-201 imaging: A new marker of triple-vessel disease. Am Heart J.

[5] Marcassa C, Galli M, Baroffio C, Campini R, Giannuzzi P (1999). Transient left ventricular dilation at quantitative stress-rest sestamibi tomography: Clinical, electrocardiographic, and angiographic correlates. J Nucl Cardiol.

[6] Chouraqui P, Rodrigues EA, Berman DS, Maddahi J (1990). Significance of dipyridamole-induced transient dilation of the left ventricle during thallium-201 scintigraphy in suspected coronary artery disease. Am J Cardiol.

[7] Kurata C, Wakabayashi Y, Shouda S, Mikami T, Tawarahara K (1996). Quantification of left ventricular size on exercise thallium-201 single-photon emission tomography. Eur J Nucl Med.

[8] Sugihara H, Shiga K, Umamoto I, Harada Y, Katahira T, Nakagawa T (1990). [Assessment of transient dilation of the left ventricular cavity in patients with hypertrophic cardiomyopathy by exercise thallium-201 scintigraphy]. Kaku Igaku.

[9] Robinson VJ, Corley JH, Marks DS, Eberhardt LW, Eubig C, Burke GJ (2000). Causes of transient dilatation of the left ventricle during myocardial perfusion imaging. AJR Am J Roentgenol.

[10] Whiting PF, Rutjes AW, Westwood ME, Mallett S, Deeks JJ, Reitsma JB (2011). QUADAS-2: A revised tool for the quality assessment of diagnostic accuracy studies. Ann Intern Med.

[11] Macaskill PGC, Deeks JJ, Harbord RM, Takwoingi Y. Cochrane handbook for systematic reviews of diagnostic test accuracy. In: Deeks JJB, Bossuyt PM, Gatsonis C (eds) The Cochrane Collaboration. 2010. http://srdta.cochrane.org/.

[12] Golzar Y, Olusanya A, Pe N, Dua SG, Golzar J, Gidea C (2015). The significance of automatically measured transient ischemic dilation in identifying severe and extensive coronary artery disease in regadenoson, single-isotope technetium-99m myocardial perfusion SPECT. J Nucl Cardiol.

[13] De Winter O, Velghe A, Van de Veire N, De Bondt P, De Buyzere M, Van De Wiele C (2005). Incremental prognostic value of combined perfusion and function assessment during myocardial gated SPECT in patients aged 75 years or older. J Nucl Cardiol.

[14] Petretta M, Acampa W, Daniele S, Petretta MP, Plaitano M, Cuocolo A (2013). Transient ischemic dilation in patients with diabetes mellitus: Prognostic value and effect on clinical outcome after coronary revascularization. Circ Cardiovasc Imaging.

[15] uz Zaman M, Fatima N, Samad A, Ishaq M, Wali A, Rehman K (2011). Predictive and prognostic values of transient ischemic dilatation of left ventricular cavity for coronary artery disease and impact of various managements on clinical outcome using technetium-99m sestamibi gated myocardial perfusion imaging. Ann Nucl Med.

[16] Lette J, Lapointe J, Waters D, Cerino M, Picard M, Gagnon A (1990). Transient left ventricular cavitary dilation during dipyridamole-thallium imaging as an indicator of severe coronary artery disease. Am J Cardiol.

[17] Abidov A, Bax JJ, Hayes SW, Hachamovitch R, Cohen I, Gerlach J (2003). Transient ischemic dilation ratio of the left ventricle is a significant predictor of future cardiac events in patients with otherwise normal myocardial perfusion SPECT. J Am Coll Cardiol.

[18] Fallahi B, Beiki D, Fard-Esfahani A, Akbarpour S, Abolhassani A, Kakhki VR (2010). The additive value of transient left ventricular dilation using two-day dipyridamole 99mTc-MIBI SPET for screening coronary artery disease in patients with otherwise normal myocardial perfusion: A comparison between diabetic and non-diabetic cases. Hell J Nucl Med.

[19] Doukky R, Frogge N, Bayissa YA, Balakrishnan G, Skelton JM, Confer K (2013). The prognostic value of transient ischemic dilatation with otherwise normal SPECT myocardial perfusion imaging: A cautionary note in patients with diabetes and coronary artery disease. J Nucl Cardiol.

[20] Mahajan N, Polavaram L, Vankayala H, Ference B, Wang Y, Ager J (2010). Diagnostic accuracy of myocardial perfusion imaging and stress echocardiography for the diagnosis of left main and triple vessel coronary artery disease: A comparative meta-analysis. Heart.

[21] Parker MW, Iskandar A, Limone B, Perugini A, Kim H, Jones C (2012). Diagnostic accuracy of cardiac positron emission tomography versus single photon emission computed tomography for coronary artery disease: A bivariate meta-analysis. Circ Cardiovasc Imaging.

[22] Sun Z, Ng KH (2012). Diagnostic value of coronary CT angiography with prospective ECG-gating in the diagnosis of coronary artery disease: A systematic review and meta-analysis. Int J Cardiovasc Imaging.

[23] Pontone G, Andreini D, Bartorelli AL, Bertella E, Mushtaq S, Annoni A (2012). Radiation dose and diagnostic accuracy of multidetector computed tomography for the detection of significant coronary artery stenoses: A meta-analysis. Int J Cardiol.

[24] Van Tosh A, Hecht S, Berger M, Roberti R, Luna E, Horowitz SF (1994). Exercise echocardiographic correlates of transient dilatation of the left ventricular cavity on exercise thallium-201 SPECT imaging. Chest.

[25] Bourque JM (2015). Contemporary relevance of TID: Based on the company it keeps. J Nucl Cardiol.

[26] Gupta A, Baradaran H, Al-Dasuqi K, Knight-Greenfield A, Giambrone AE, Delgado D (2016). Gadolinium enhancement in intracranial atherosclerotic plaque and ischemic stroke: A systematic review and meta-analysis. J Am Heart Assoc.

[27] Rischpler C, Higuchi T, Fukushima K, Javadi MS, Merrill J, Nekolla SG (2012). Transient ischemic dilation ratio in 82Rb PET myocardial perfusion imaging: Normal values and significance as a diagnostic and prognostic marker. J Nucl Med.

[28] Xu Y, Arsanjani R, Clond M, Hyun M, Lemley M, Fish M (2012). Transient ischemic dilation for coronary artery disease in quantitative analysis of same-day sestamibi myocardial perfusion SPECT. J Nucl Cardiol.

[29] Kinoshita N, Sugihara H, Adachi Y, Nakamura T, Azuma A, Kohno Y (2002). Assessment of transient left ventricular dilatation on rest and exercise on Tc-99m tetrofosmin myocardial SPECT. Clin Nucl Med.

[30] Petretta M, Acampa W, Daniele S, Petretta MP, Nappi C, Assante R (2013). Transient ischemic dilation in SPECT myocardial perfusion imaging for prediction of severe coronary artery disease in diabetic patients. J Nucl Cardiol.

[31] Emmett L, Van Gaal WJ, Magee M, Bass S, Ali O, Freedman SB (2008). Prospective evaluation of the impact of diabetes and left ventricular hypertrophy on the relationship between ischemia and transient ischemic dilation of the left ventricle on single-day adenosine Tc-99m myocardial perfusion imaging. J Nucl Cardiol.

[32] Emmett L, Magee M, Freedman SB, Van der Wall H, Bush V, Trieu J (2005). The role of left ventricular hypertrophy and diabetes in the presence of transient ischemic dilation of the left ventricle on myocardial perfusion SPECT images. J Nucl Med.

[33] Mazzanti M, Germano G, Kiat H, Kavanagh PB, Alexanderson E, Friedman JD (1996). Identification of severe and extensive coronary artery disease by automatic measurement of transient ischemic dilation of the left ventricle in dual-isotope myocardial perfusion SPECT. J Am Coll Cardiol.

[34] Abidov A, Bax JJ, Hayes SW, Cohen I, Nishina H, Yoda S (2004). Integration of automatically measured transient ischemic dilation ratio into interpretation of adenosine stress myocardial perfusion SPECT for detection of severe and extensive CAD. J Nucl Med.

[35] Lette J, Bertrand C, Gossard D, Ruscito O, Cerino M, McNamara D (1995). Long-term risk stratification with dipyridamole imaging. Am Heart J.

[36] Thomas GS, Miyamoto MI, Morello AP, Majmundar H, Thomas JJ, Sampson CH (2004). Technetium 99m sestamibi myocardial perfusion imaging predicts clinical outcome in the community outpatient setting. The Nuclear Utility in the Community (NUC) Study. J Am Coll Cardiol.

