# Supplementary material for: Diagnostic and prognostic significance of transient ischemic dilation (TID) in myocardial perfusion imaging: A systematic review and meta-analysis
Source: J Nucl Cardiol. 2017 Sep 25;25(3):724–37. doi: 10.1007/s12350-017-1040-7 (PMC5966496; doi:10.1007/s12350-017-1040-7)
Supplement: Supplementary file 1 — Supplementary material 1 (DOCX 137 kb) [file 12350_2017_1040_MOESM1_ESM.docx]

ONLINE Table A. List of excluded full-text studies (Group Z)

| Study | Reason for exclusion |
| --- | --- |
| Iskandrian^1^ | No angiographic data |
| Smelley^2^ | No angiographic data  Included low risk patients only |
| Rivero^3^ | No angiographic data |
| Hansen^4^ | No angiographic data |
| Emmett^5^ | Incomplete data , Reported patients with severe Coronary artery disease only, no angiography negative group |
| Hung^6^ | No angiographic data |
| Hansen^7^ | No angiographic data |
| Kakhki^8^ | No angiographic data |
| McClellan^9^ | No TID value mentioned |
| Bestetti^10^ | No TID value mentioned |
| Mandour^11^ | No angiographic data |
| Heston^12^ | No angiographic data |
| Demir^13^ | No angiographic data |
| Valdiviezo^14^ | Used another method for TID calculation (TID index); Also reported only aggregate data |
| Azambuja Gonzalez^15^ | No angiographic data |
| Bestetti^16^ | Done in patients with Left ventricular dysfunction and fixed perfusion defects |
| Brodov^17^ | Incomplete data, used to assess the diagnostic accuracy of TID in attenuation corrected versus non attenuation corrected studies |
| Ahlberg^18^ | Used visual assessment of TID, no quantitative values |
| Fukuda^19^ | Included the myocardium in TID quantitative assessment |
| Daou^20^ | Duplicate data, has the same patient population in another study^21^ |

ONLINE Table B. Characteristics of studies reporting only aggregate data and unable to include in meta-analysis (Group C)

| **Study** | **Stress / Tracer** | **TID Ratio (Quant or Qual)** | **# SPECT vs. Coronary angiography or CTA** | **Cardiac risk factors** | **Cardiac**  **characteristics** |
| --- | --- | --- | --- | --- | --- |
| Hida^22^  63±10 years  90% men | Exercise  Tc 99 m | 0.99 (Quant) | SPECT: 278 pts  Angiography or CTA: 278 pts | Smoking 49%, HTN 77%, DM 52%, Dyslipidemia 75% | MI 43%  Prior PCI 41% |
| Romanens^23^  55±19 years  91% men | Exercise  Tc 99 m | 1.18 (Quant) | SPECT: 120 pts  Angiography or CTA: 120 pts | N/A | Angina 50%  MI 55% |
| Peace^24^  N/A | Exercise  Tc 99 m | 1.1 (Quant) | SPECT: 50 pts  Angiography or CTA: 50 pts | N/A |  |
| Hida^25^  69±10 years  69% men | Adenosine  Tc 99 m | 1.11 (Quant) | SPECT: 271 pts  Angiography or CTA: 271 pts | Smoking 54%, HTN 78%, DM 45%, Dyslipidemia 59% | MI 26%  Prior PCI 27% |
| Daou^21^  60±10 years | Exercise  Tl 201 | 1.25 (Quant) | SPECT: 310 pts  Angiography or CTA: 310 pts | N/A | MI 67% |
| Katz^26^  67 years  48% men | Regadenoson  Dual | 1.39 (Quant) | SPECT: 195 pts  Angiography or CTA: 195 pts | Smoking 36%, HTN 77%, DM 45%, Dyslipidemia 63% |  |
| Berman^27^  69±12 years  78% men | Ex & Pharm  Dual | 1.22 Ex (Quant)  1.36 Ph (Quant) | SPECT: 101 pts  Angiography or CTA: 101 pts | Smoking 6%, HTN 69%, DM 33%, Dyslipidemia 52% | Angina 50% |
| Aldhilan^28^  66.7±11 years  71% men | Ex & Pharm  Tc 99 m | 1.19 (Quant) | SPECT: 52 pts  Angiography or CTA: 44 pts | N/A |  |
| Shi^29^  63±11 years  49% men | Pharm  Rb 82 | 1.15 (Quant) | SPECT: 95 pts  Angiography or CTA: 65 pts | N/A |  |
| Nakazato^30^  70±12 years  42% men | Pharm  Rb 82 | 1.28 (Quant) | SPECT: 125 pts  Angiography or CTA: 83 pts | Smoking 10%, HTN 54%,, DM 25%, Dyslipidemia 42% | Angina 6% |

EX: Exercise, Pharm: Pharmacologic, Quant: Quantitative, Qual: Qualitative, CTA: CT angiography, HTN: Hypertension,

**REFERENCE LIST**

1. Iskandrian AS, Heo J, Nguyen T, Lyons E, Paugh E. Left ventricular dilatation and pulmonary thallium uptake after single-photon emission computer tomography using thallium-201 during adenosine-induced coronary hyperemia. *Am J Cardiol.* 1990;66(10):807-811.

2. Smelley MP, Virnich DE, Williams KA, Ward RP. A hypertensive response to exercise is associated with transient ischemic dilation on myocardial perfusion SPECT imaging. *J Nucl Cardiol.* 2007;14(4):537-543.

3. Rivero A, Santana C, Folks RD, et al. Attenuation correction reveals gender-related differences in the normal values of transient ischemic dilation index in rest-exercise stress sestamibi myocardial perfusion imaging. *J Nucl Cardiol.* 2006;13(3):338-344.

4. Hansen CL, Sangrigoli R, Nkadi E, Kramer M. Comparison of pulmonary uptake with transient cavity dilation after exercise thallium-201 perfusion imaging. *J Am Coll Cardiol.* 1999;33(5):1323-1327.

5. Emmett L, Ng A, Ha L, et al. Comparative assessment of rest and post-stress left ventricular volumes and left ventricular ejection fraction on gated myocardial perfusion imaging (MPI) and echocardiography in patients with transient ischaemic dilation on adenosine MPI: myocardial stunning or subendocardial hypoperfusion? *J Nucl Cardiol.* 2012;19(4):735-742.

6. Hung GU, Lee KW, Chen CP, Lin WY, Yang KT. Relationship of transient ischemic dilation in dipyridamole myocardial perfusion imaging and stress-induced changes of functional parameters evaluated by Tl-201 gated SPECT. *J Nucl Cardiol.* 2005;12(3):268-275.

7. Hansen CL, Cen P, Sanchez B, Robinson R. Comparison of pulmonary uptake with transient cavity dilation after dipyridamole Tl-201 perfusion imaging. *J Nucl Cardiol.* 2002;9(1):47-51.

8. Kakhki VR, Sadeghi R, Zakavi SR. Assessment of transient left ventricular dilation ratio via 2-day dipyridamole Tc-99m sestamibi nongated myocardial perfusion imaging. *J Nucl Cardiol.* 2007;14(4):529-536.

9. McClellan JR, Travin MI, Herman SD, et al. Prognostic importance of scintigraphic left ventricular cavity dilation during intravenous dipyridamole technetium-99m sestamibi myocardial tomographic imaging in predicting coronary events. *Am J Cardiol.* 1997;79(5):600-605.

10. Bestetti A, Di Leo C, Alessi A, Triulzi A, Tagliabue L, Tarolo GL. Post-stress end-systolic left ventricular dilation: a marker of endocardial post-ischemic stunning. *Nucl Med Commun.* 2001;22(6):685-693.

11. Mandour Ali MA, Bourque JM, Allam AH, Beller GA, Watson DD. The prevalence and predictive accuracy of quantitatively defined transient ischemic dilation of the left ventricle on otherwise normal SPECT myocardial perfusion imaging studies. *J Nucl Cardiol.* 2011;18(6):1036-1043.

12. Heston TF, Sigg DM. Quantifying transient ischemic dilation using gated SPECT. *J Nucl Med.* 2005;46(12):1990-1996.

13. Demir H, Tan YZ, Isgoren S, et al. Comparison of exercise and pharmacological stress gated SPECT in detecting transient left ventricular dysfunction. *Ann Nucl Med.* 2008;22(5):403-409.

14. Valdiviezo C, Motivala AA, Hachamovitch R, et al. The significance of transient ischemic dilation in the setting of otherwise normal SPECT radionuclide myocardial perfusion images. *J Nucl Cardiol.* 2011;18(2):220-229.

15. Azambuja Gonzalez MB, Azambuja RA, Bodanese LC. Quantification of left ventricular dilatation in myocardial perfusion scintigraphy. *Arq Bras Cardiol.* 2011;96(5):363-368.

16. Bestetti A, Bigi R, Terranova P, Lombardi F, Fiorentini C. Prognostic implications of stress-induced transient ischemic dilation of the left ventricle in patients with systolic dysfunction and fixed perfusion defects. *Int J Cardiol.* 2010;140(3):323-327.

17. Brodov Y, Frenkel A, Chouraqui P, et al. Influence of attenuation correction on transient left ventricular dilation in dual isotope myocardial perfusion imaging in patients with known or suspected coronary artery disease. *Am J Cardiol.* 2012;110(1):57-61.

18. Ahlberg AW, Baghdasarian SB, Athar H, et al. Symptom-limited exercise combined with dipyridamole stress: prognostic value in assessment of known or suspected coronary artery disease by use of gated SPECT imaging. *J Nucl Cardiol.* 2008;15(1):42-56.

19. Fukuda H, Moroi M. Prediction of cardiac events in patients with transient left ventricle dilation on stress myocardial perfusion SPECT images. *Circ J.* 2005;69(10):1223-1229.

20. Daou D, Coaguila C, Delahaye N, Houzet F, Lebtahi R, Le Guludec D. Discordance between exercise SPECT lung Tl-201 uptake and left ventricular transient ischemic dilation in patients with CAD. *J Nucl Cardiol.* 2004;11(1):53-61.

21. Daou D, Delahaye N, Vilain D, Lebtahi R, Faraggi M, Le Guludec D. Identification of extensive coronary artery disease: incremental value of exercise Tl-201 SPECT to clinical and stress test variables. *J Nucl Cardiol.* 2002;9(2):161-168.

22. Hida S, Chikamori T, Tanaka H, et al. Diagnostic value of left ventricular dyssynchrony after exercise and at rest in the detection of multivessel coronary artery disease on single-photon emission computed tomography. *Circ J.* 2012;76(8):1942-1952.

23. Romanens M, Gradel C, Saner H, Pfisterer M. Comparison of 99mTc-sestamibi lung/heart ratio, transient ischaemic dilation and perfusion defect size for the identification of severe and extensive coronary artery disease. *Eur J Nucl Med.* 2001;28(7):907-910.

24. Peace RA, McKiddie FI, Staff RT, Gemmell HG. Comparison of methods for quantification of transient ischaemic dilation in myocardial perfusion SPET. *Nucl Med Commun.* 2000;21(10):971-976.

25. Hida S, Chikamori T, Tanaka H, et al. Postischemic myocardial stunning is superior to transient ischemic dilation for detecting multivessel coronary artery disease. *Circ J.* 2012;76(2):430-438.

26. Katz JS, Ruisi M, Giedd KN, Rachko M. Assessment of transient ischemic dilation (TID) ratio in gated SPECT myocardial perfusion imaging (MPI) using regadenoson, a new agent for pharmacologic stress testing. *J Nucl Cardiol.* 2012;19(4):727-734.

27. Berman DS, Kang X, Slomka PJ, et al. Underestimation of extent of ischemia by gated SPECT myocardial perfusion imaging in patients with left main coronary artery disease. *J Nucl Cardiol.* 2007;14(4):521-528.

28. Aldhilan A, Syed GM, Suleiman I, Al Zaibag M, Fielding H. Correlation between transient ischemic dilatation (TID) and coronary artery disease in Saudi male patients. *J Saudi Heart Assoc.* 2014;26(1):1-5.

29. Shi H, Santana CA, Rivero A, et al. Normal values and prospective validation of transient ischaemic dilation index in 82Rb PET myocardial perfusion imaging. *Nucl Med Commun.* 2007;28(11):859-863.

30. Nakazato R, Berman DS, Dey D, et al. Automated quantitative Rb-82 3D PET/CT myocardial perfusion imaging: normal limits and correlation with invasive coronary angiography. *J Nucl Cardiol.* 2012;19(2):265-276.
