# Supplementary material for: Diagnostic and prognostic significance of transient ischemic dilation (TID) in myocardial perfusion imaging: A systematic review and meta-analysis
Source: J Nucl Cardiol. 2017 Sep 25;25(3):724–37. doi: 10.1007/s12350-017-1040-7 (PMC5966496; doi:10.1007/s12350-017-1040-7)
Supplement: Supplementary file 2 — Supplementary material 2 (PPTX 4413 kb) [file 12350_2017_1040_MOESM2_ESM.pptx]

## Slide 1
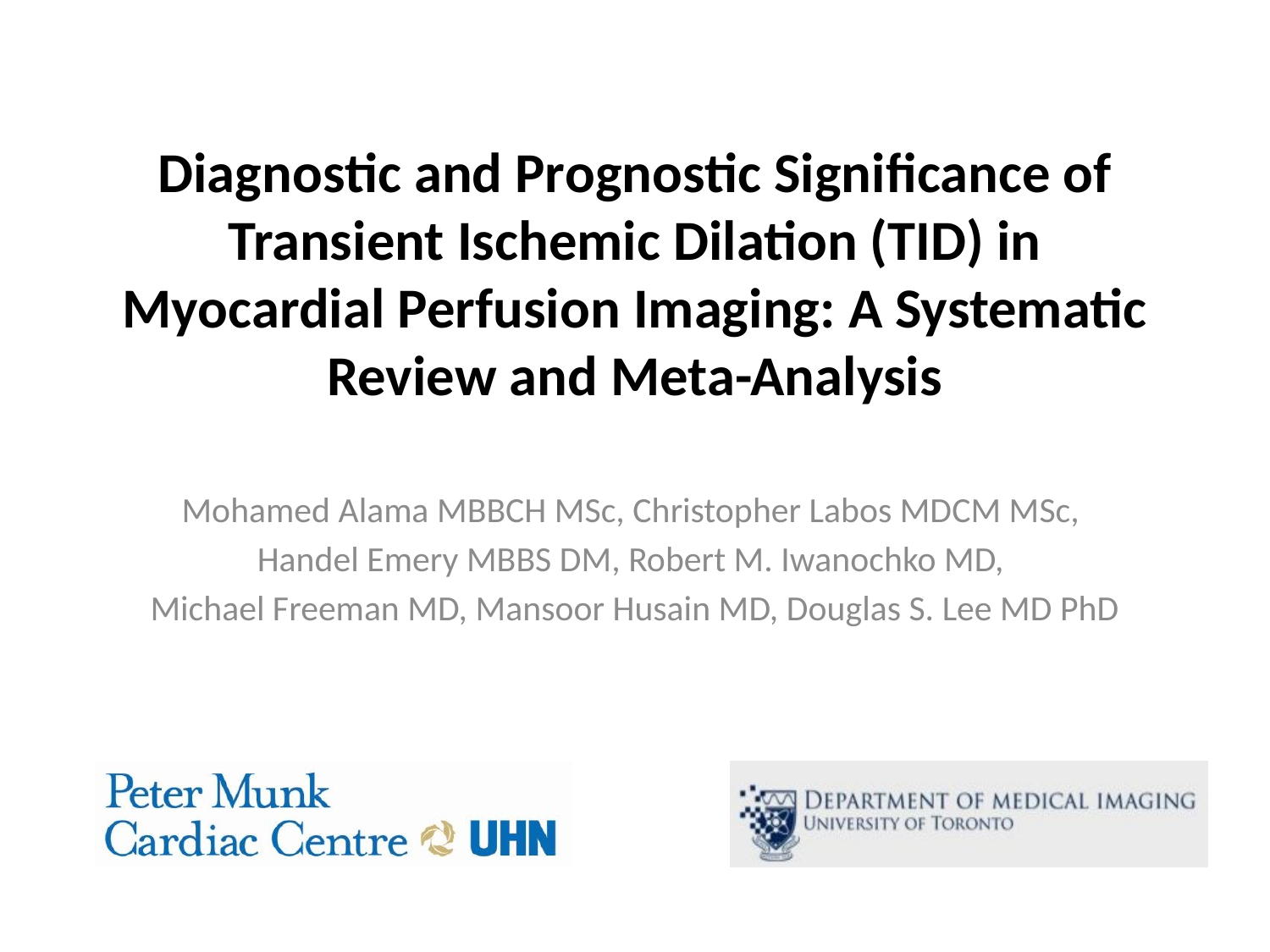

# Diagnostic and Prognostic Significance of Transient Ischemic Dilation (TID) in Myocardial Perfusion Imaging: A Systematic Review and Meta-Analysis
Mohamed Alama MBBCH MSc, Christopher Labos MDCM MSc,
Handel Emery MBBS DM, Robert M. Iwanochko MD,
Michael Freeman MD, Mansoor Husain MD, Douglas S. Lee MD PhD

## Slide 2
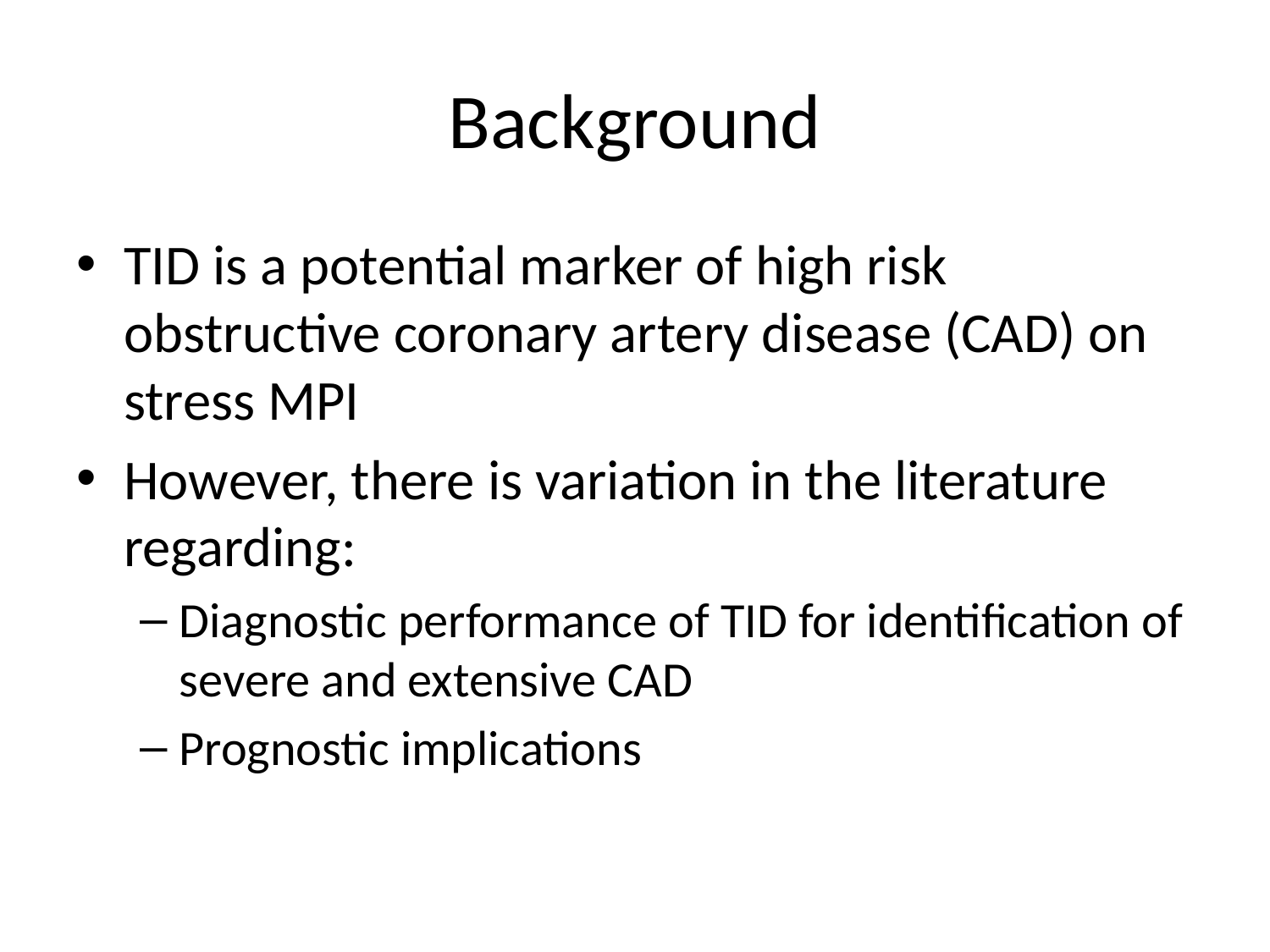

# Background
TID is a potential marker of high risk obstructive coronary artery disease (CAD) on stress MPI
However, there is variation in the literature regarding:
Diagnostic performance of TID for identification of severe and extensive CAD
Prognostic implications

## Slide 3
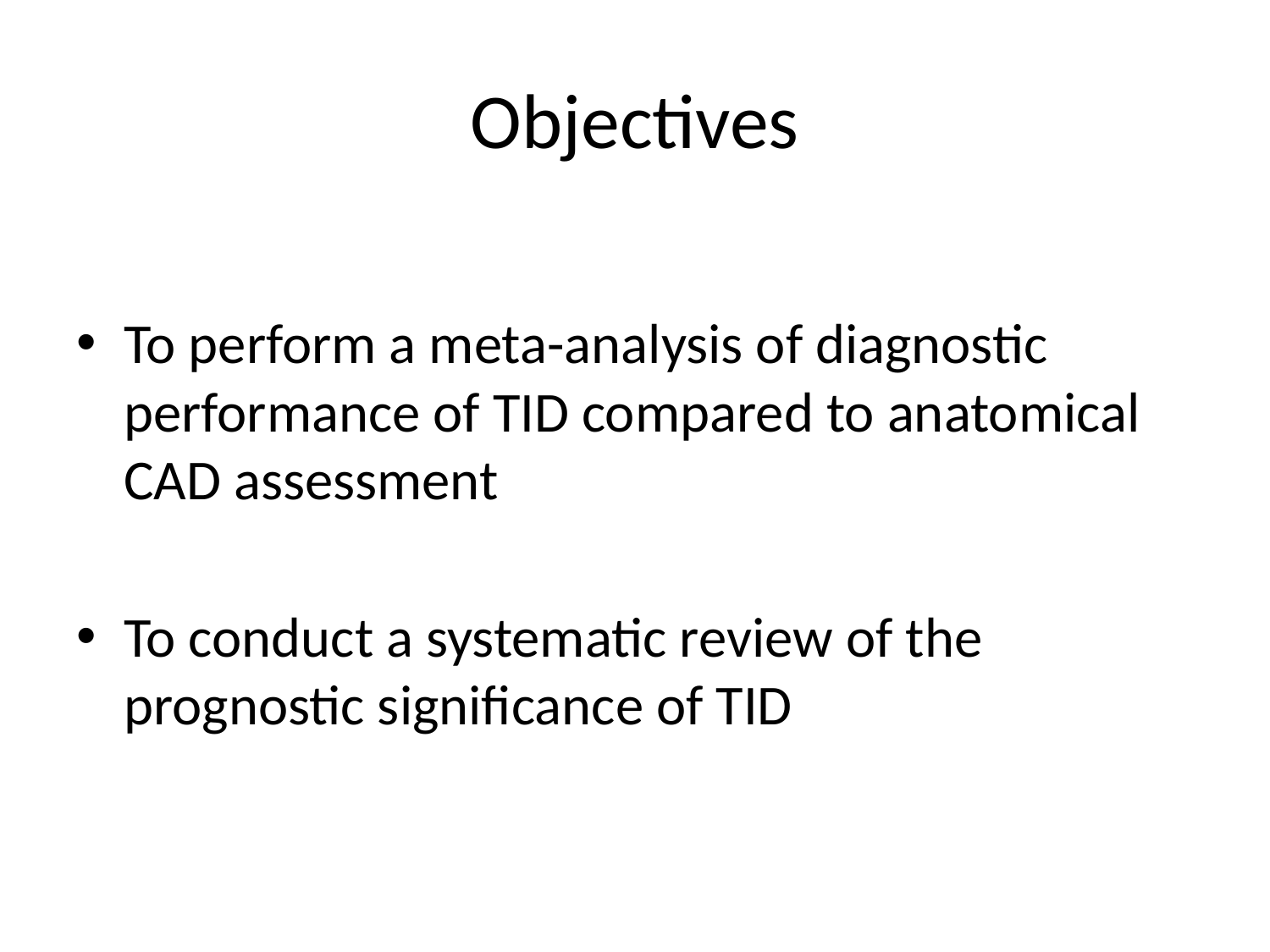

# Objectives
To perform a meta-analysis of diagnostic performance of TID compared to anatomical CAD assessment
To conduct a systematic review of the prognostic significance of TID

## Slide 4
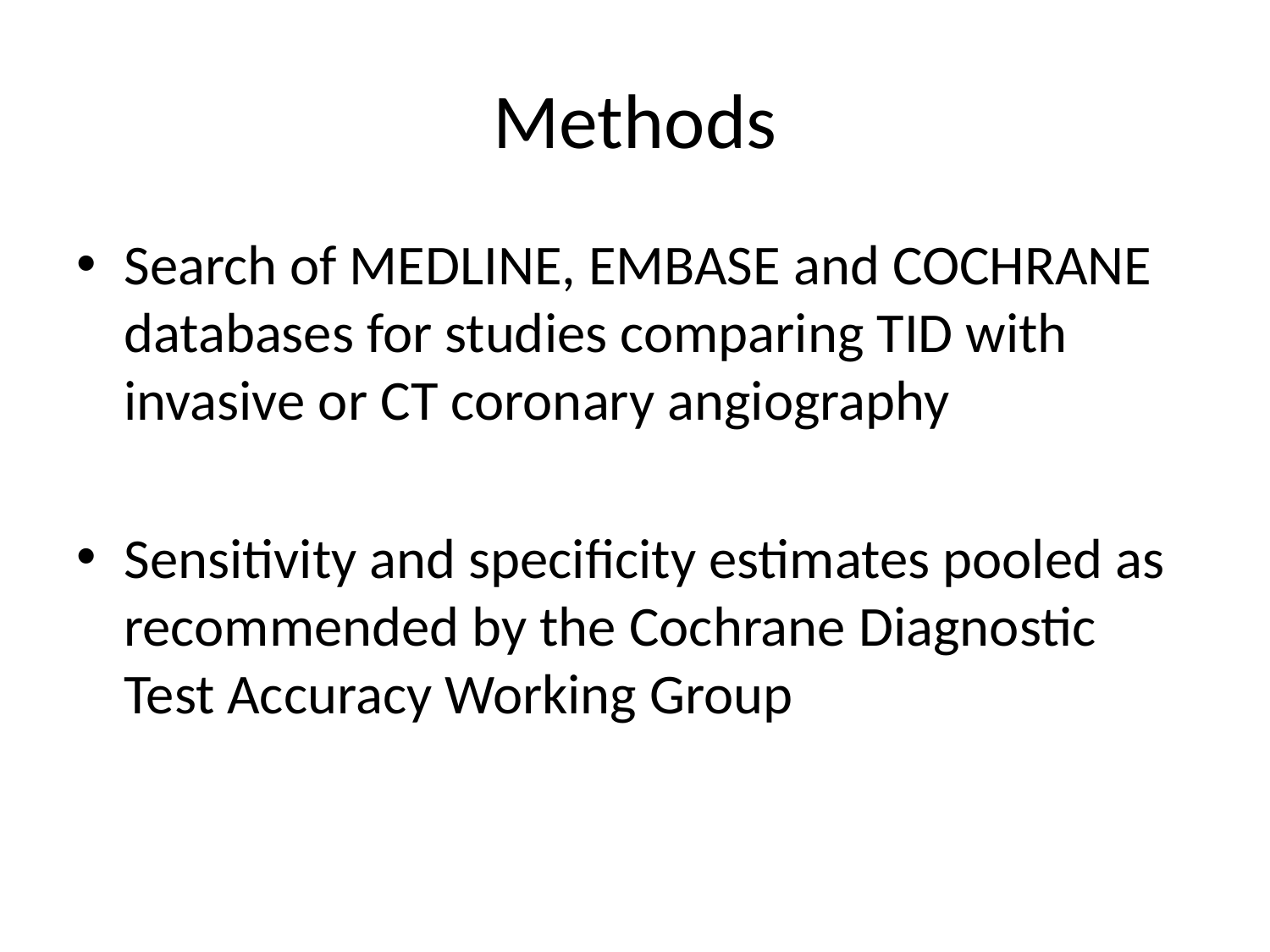

# Methods
Search of MEDLINE, EMBASE and COCHRANE databases for studies comparing TID with invasive or CT coronary angiography
Sensitivity and specificity estimates pooled as recommended by the Cochrane Diagnostic Test Accuracy Working Group

## Slide 5
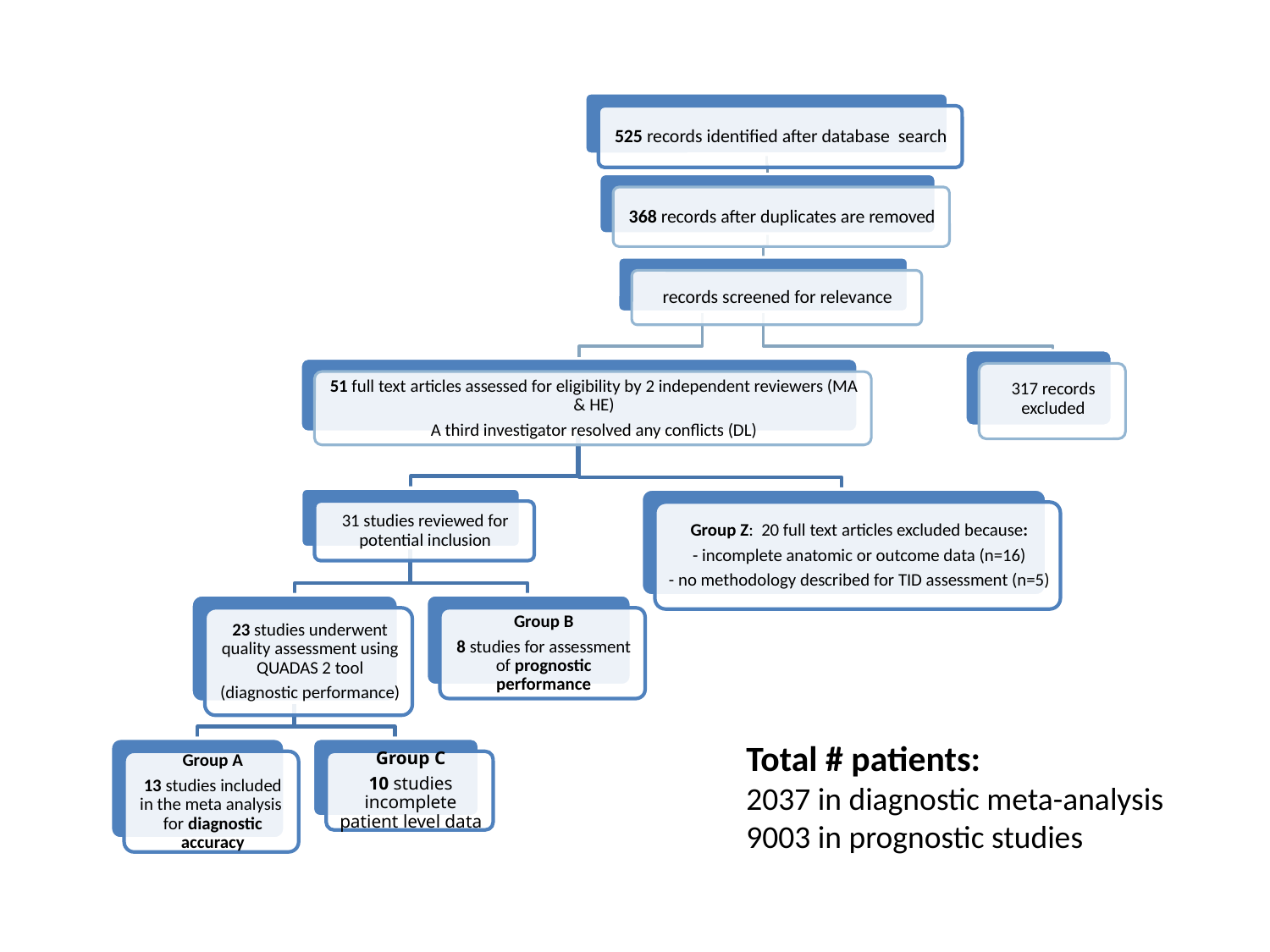

Total # patients:
2037 in diagnostic meta-analysis
9003 in prognostic studies

## Slide 6
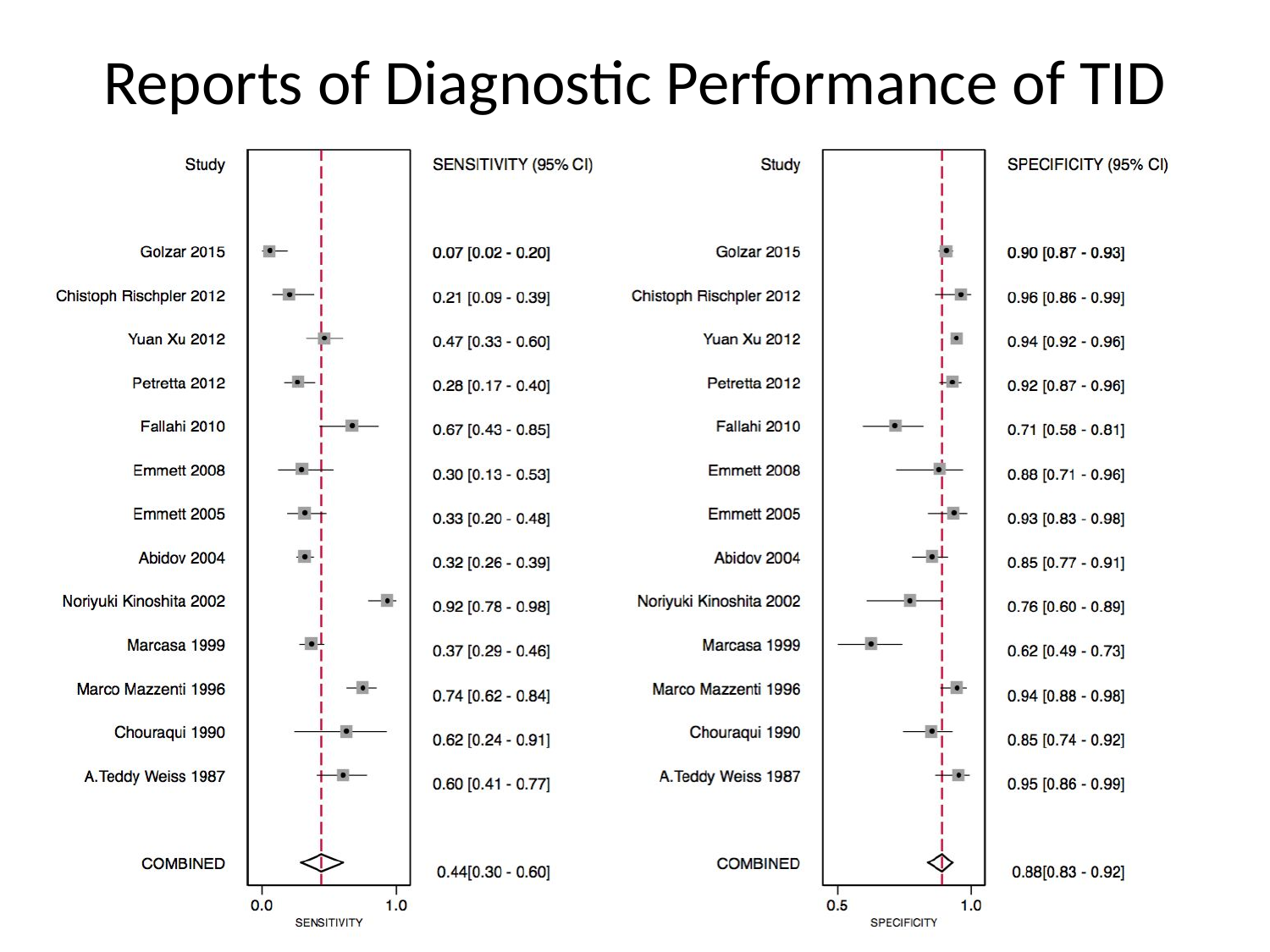

# Reports of Diagnostic Performance of TID

## Slide 7
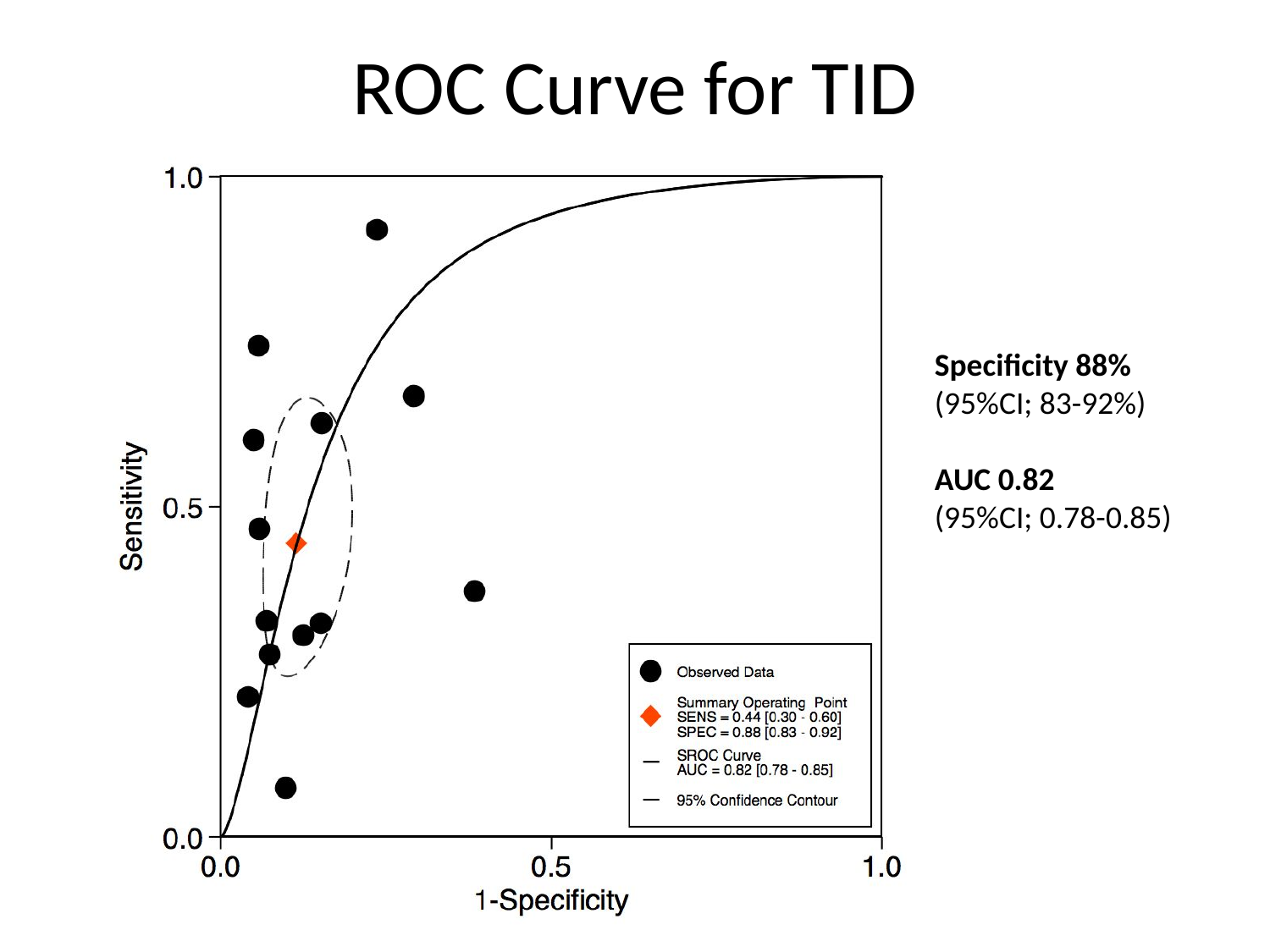

# ROC Curve for TID
Specificity 88%
(95%CI; 83-92%)
AUC 0.82
(95%CI; 0.78-0.85)

## Slide 8
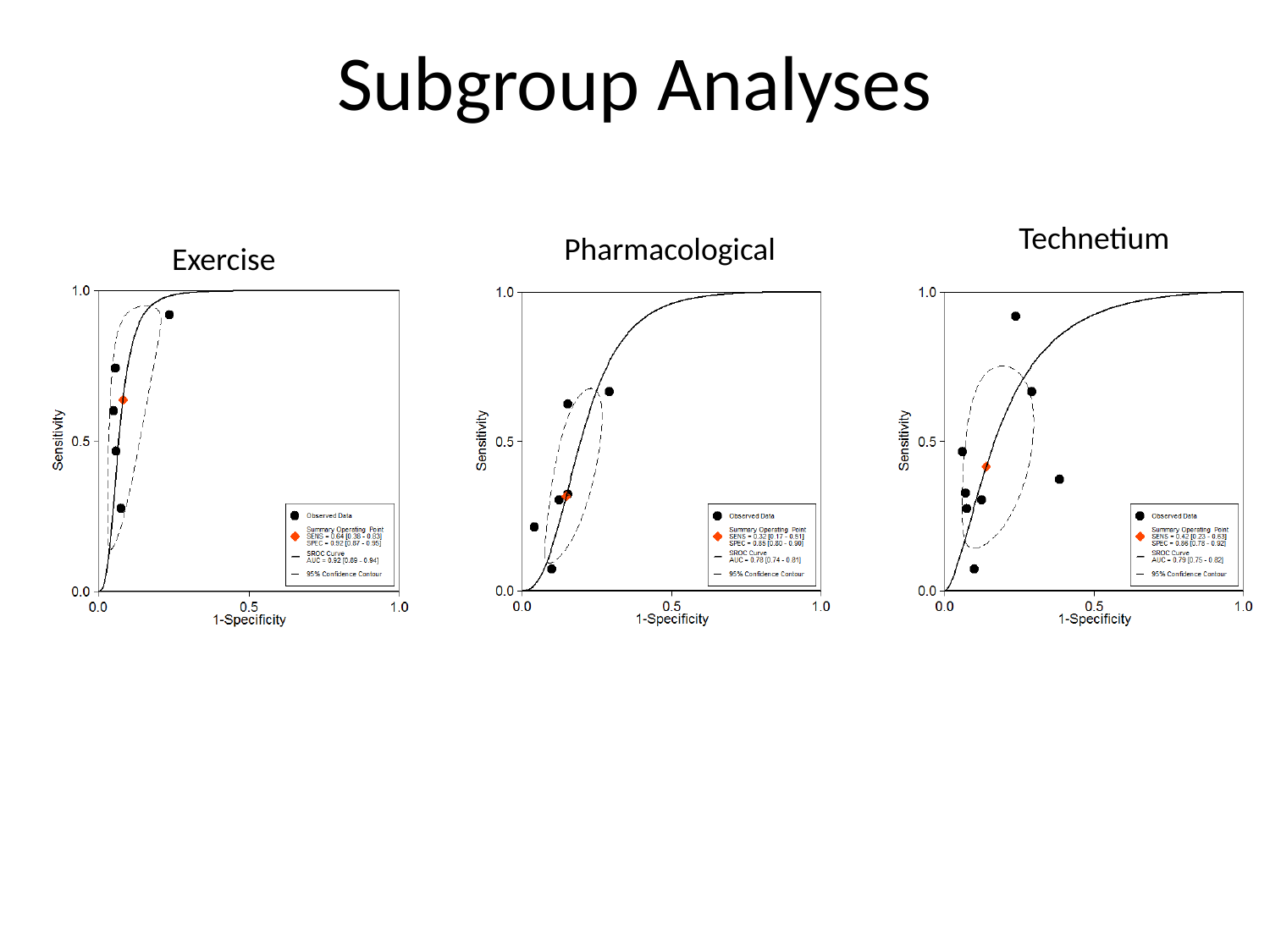

# Subgroup Analyses
Technetium
Pharmacological
Exercise

## Slide 9
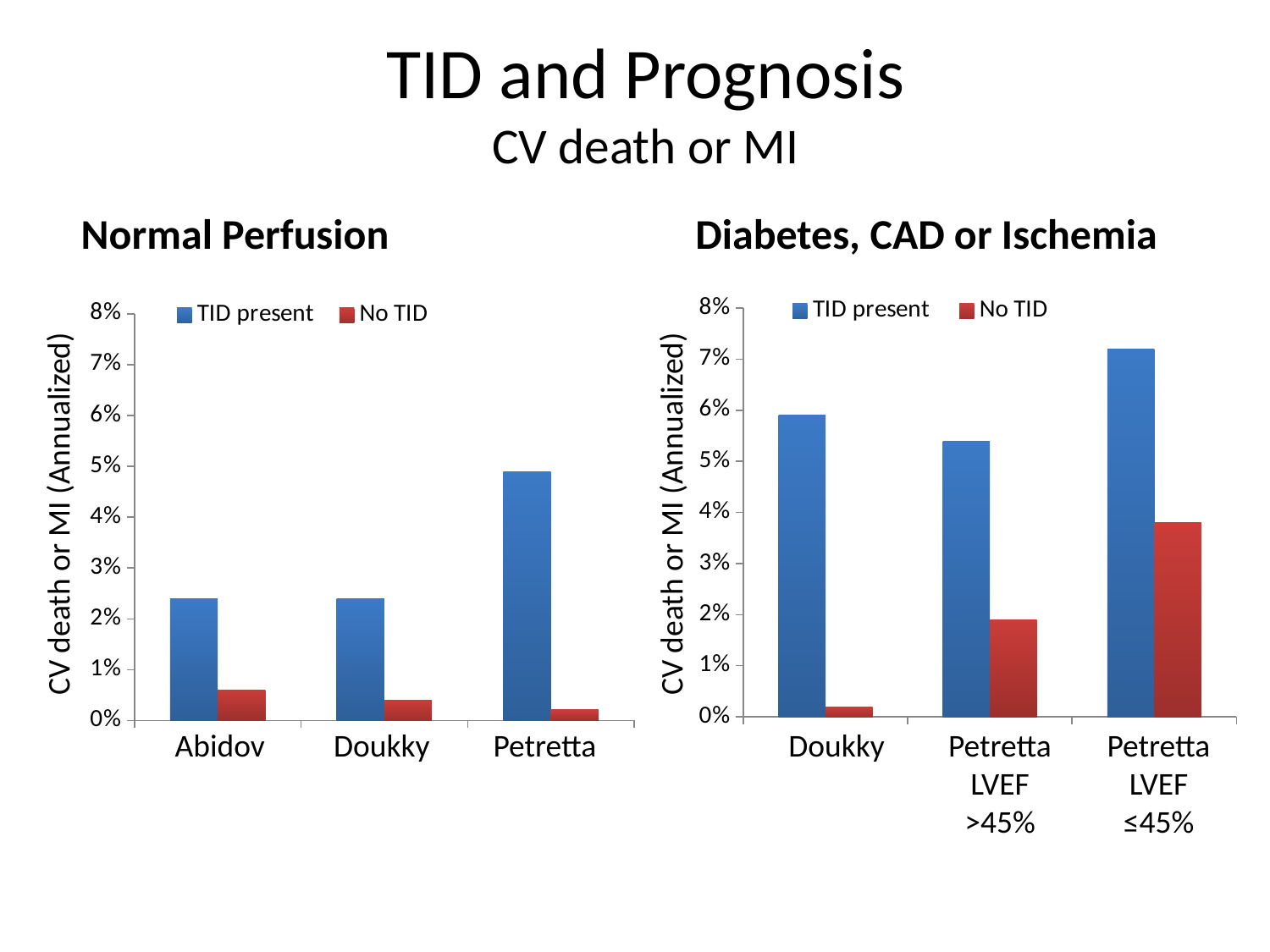

# TID and Prognosis CV death or MI
Normal Perfusion
Diabetes, CAD or Ischemia
### Chart
| Category | TID present | No TID |
|---|---|---|CV death or MI (Annualized)
Abidov
Doukky
Petretta
Doukky
Petretta
LVEF
>45%
Petretta
LVEF
≤45%
### Chart
| Category | TID present | No TID |
|---|---|---|CV death or MI (Annualized)

## Slide 10
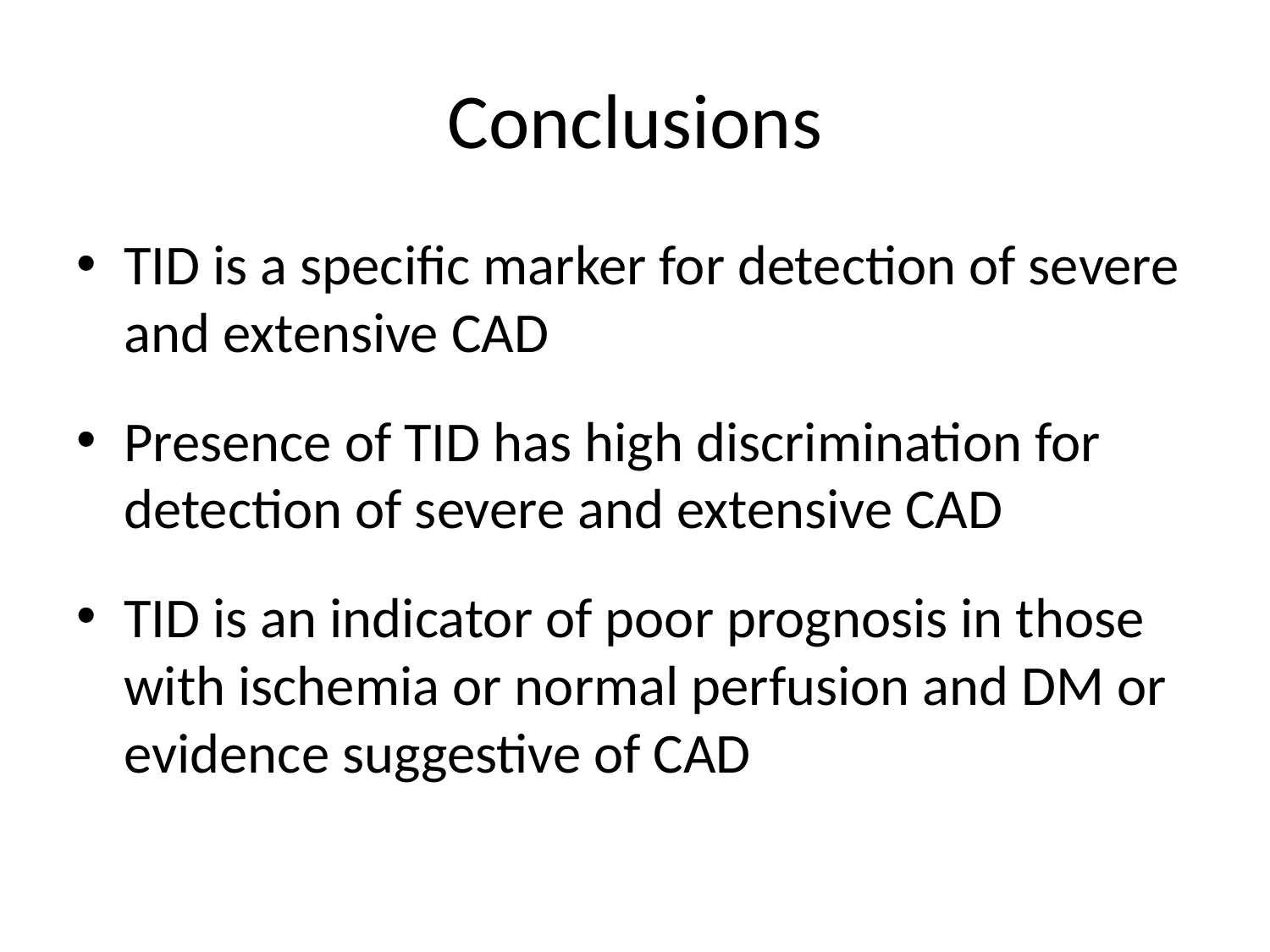

# Conclusions
TID is a specific marker for detection of severe and extensive CAD
Presence of TID has high discrimination for detection of severe and extensive CAD
TID is an indicator of poor prognosis in those with ischemia or normal perfusion and DM or evidence suggestive of CAD
